# Complete genome sequence analysis of the thermoacidophilic verrucomicrobial methanotroph “*Candidatus* Methylacidiphilum kamchatkense” strain Kam1 and comparison with its closest relatives

**DOI:** 10.1186/s12864-019-5995-4

**Published:** 2019-08-09

**Authors:** Thomas Kruse, Chandini Murarilal Ratnadevi, Helge-André Erikstad, Nils-Kåre Birkeland

**Affiliations:** Department of Biological Sciences, University of Bergen, P.O. Box 7803, 5020 Bergen, Norway

**Keywords:** “*Ca*. Methylacidiphilum kamchatkense” strain Kam1, Full genome, Methane oxidation, Thermoacidophile, Methanotroph

## Abstract

**Background:**

The candidate genus “Methylacidiphilum” comprises thermoacidophilic aerobic methane oxidizers belonging to the Verrucomicrobia phylum. These are the first described non-proteobacterial aerobic methane oxidizers. The genes *pmoCAB,* encoding the particulate methane monooxygenase do not originate from horizontal gene transfer from proteobacteria. Instead, the “*Ca*. Methylacidiphilum” and the sister genus “*Ca*. Methylacidimicrobium” represent a novel and hitherto understudied evolutionary lineage of aerobic methane oxidizers. Obtaining and comparing the full genome sequences is an important step towards understanding the evolution and physiology of this novel group of organisms.

**Results:**

Here we present the closed genome of “*Ca*. Methylacidiphilum kamchatkense” strain Kam1 and a comparison with the genomes of its two closest relatives “*Ca*. Methylacidiphilum fumariolicum” strain SolV and “*Ca*. Methylacidiphilum infernorum” strain V4. The genome consists of a single 2,2 Mbp chromosome with 2119 predicted protein coding sequences. Genome analysis showed that the majority of the genes connected with metabolic traits described for one member of “*Ca*. Methylacidiphilum” is conserved between all three genomes. All three strains encode class I CRISPR-cas systems. The average nucleotide identity between “*Ca*. M. kamchatkense” strain Kam1 and strains SolV and V4 is ≤95% showing that they should be regarded as separate species. Whole genome comparison revealed a high degree of synteny between the genomes of strains Kam1 and SolV. In contrast, comparison of the genomes of strains Kam1 and V4 revealed a number of rearrangements. There are large differences in the numbers of transposable elements found in the genomes of the three strains with 12, 37 and 80 transposable elements in the genomes of strains Kam1, V4 and SolV respectively. Genomic rearrangements and the activity of transposable elements explain much of the genomic differences between strains. For example, a type 1h uptake hydrogenase is conserved between strains Kam1 and SolV but seems to have been lost from strain V4 due to genomic rearrangements.

**Conclusions:**

Comparing three closed genomes of “*Ca*. Methylacidiphilum” spp. has given new insights into the evolution of these organisms and revealed large differences in numbers of transposable elements between strains, the activity of these explains much of the genomic differences between strains.

**Electronic supplementary material:**

The online version of this article (10.1186/s12864-019-5995-4) contains supplementary material, which is available to authorized users.

## Background

Interest in methane oxidizing bacteria has been fueled by the fact that methane on one hand; is estimated to be a 25 times stronger greenhouse gas than CO_2_, seen over a century [1]; on the other hand methane is an inexpensive starting material for biochemical synthesis of high value products [2]. The majority of all described aerobic methane oxidizing bacteria belongs to the alpha- or gamma-proteobacteria. Members of the verrucomicrobia and the intra-aerobic members of the candidate phylum NC10 are notable exceptions [3, 4]. In 2007–8 three groups independently described the isolation of methanotrophic thermoacidophilic verrucomicrobia, strains Kam1, SolV and V4 from acidic geothermal sites in Russia, Italy and New Zealand respectively [5–7]. Based on the low (< 83%) 16S rRNA gene similarity to other verrucomicrobia it was suggested that they represent a new order, with the proposed order/family/genus names “*Candidatus* (*Ca.*) Methylacidiphilales/Methylacidiphilacea/Methylacidiphilum” [3]. Average nucleotide identity (ANI) comparison showed that strains SolV and V4 constitute two different species with the proposed species names “*Ca*. Methylacidiphilum fumariolicum” and “*Ca*. Methylacidiphilum infernorum” respectively. Due to lack of full genome information for strain Kam1, the ANI between this strain and SolV/V4 could not be determined. Despite the fact that the 16S rRNA genes of strains Kam1 and SolV are 99.7% identical, it was proposed that the former also represents a novel species, “*Ca*. Methylacidiphilum kamchatkense” [3]*.* For the remainder of this paper we will predominately use the strain designations Kam1, SolV and V4 to refer to the different representatives. Additional acidophilic, thermophilic and mesophilic, verrucomicrobial methanotrophs have been isolated from acidic environments demonstrating that these may be widespread [8, 9].

Members of the “*Ca*. Methylacidiphilaceae” are gram negative, non-motile moderate thermoacidophiles with a growth optimum at 55–60 °C and capable of growing at a wide pH range of 0.8 to 6. Their genomes do not encode a soluble methane monooxygenase (sMMO), but contains three *pmoCAB* operons, coding for the particulate methane monooxygenase (pMMO). Strain Kam1 encodes an additional unique and truncated *pmoCA* cluster that is not present in strains SolV and V4 [3, 10]. The *pmo* genes from “*Ca*. Methylacidiphilum spp.” form a distinct phylogenetic group, separate from their proteobacterial counterparts, showing that they do not originate from a recent horizontal gene transfer [3].

The “*Ca*. Methylacidiphilaceae” possess a range of traits clearly separating them from their proteobacterial counterparts. They are the most acidophilic methanotrophs described and grow autotrophically fixing CO_2_ via the Calvin Benson Bassham (CBB) cycle. Unlike proteobacterial methanotrophs that assimilate carbon via either; the RuMP cycle (group I) or the serine cycle (Group II) [2, 11]. Finally their methanol dehydrogenases are of the XoxF type containing lanthanides, a group of rare earth elements, instead of calcium in the active site [12]. Closed genomes are available for strains SolV and V4, whereas a multi-contig draft genome has been published for strain Kam1 [13–15]. Genome analysis has given a deeper understanding of the metabolism of strains SolV and V4. For example, a number of genes predicted to encode hydrogenases were observed in the genomes of strains SolV and V4 [13, 14]. This led to the speculation that they might be capable of autotrophic growth on H_2_, O_2_ and CO_2_, a metabolic trait that later has been verified experimentally for strain SolV and the recently described “*Ca*. Methylacidiphilum sp. strain RTK17.1” [9, 16].

Comparison of the closed genomes of strains SolV and V4, revealed large numbers of genomic rearrangements, hampering detailed comparison of genome architecture. Comparisons of the protein encoding genes showed that 64,3% of the protein encoding genes from SolV have more than 50% amino acid identity to genes from strain V4 [14].

We here present the closed genome of “*Ca*. Methylacidiphilum kamchatkense” Kam1, and compare it with the two previously published genomes from the “*Ca*. Methylacidiphilum” genus. We use genome comparisons as backbone for discussion of phylogeny and genome architecture of strains Kam1, SolV and V4. Finally, we link genome analysis with metabolic and physiological traits reported in the literature.

## Results

We employed PacBio RS technology in order to obtain the complete genome sequence of “*Ca*. Methylacidiphilum kamchatkense” strain Kam1. After quality, checks of the reads we ended with 94,711 reads with an average read length of 14,734 and N50 read length of 21,352 bp with an average coverage of 489.

After assembly and manual trimming of overlapping ends, we obtained the full genome of strain Kam1. The closed genome of Kam1 consists of a single circular 2,202,032 bp chromosome, which is approximately 85 and 275 kbp less than the genomes of V4 and SolV, respectively (Table 1). We did not identify any extrachromosomal genetic elements in strain Kam1. The annotation identified 2119 coding sequences hereof 1589 with a predicted function (Table 1). The genome encodes a single 16S rRNA operon and four *pmoA* genes, the latter feature distinguishes Kam1 from SolV and V4 that harbor three *pmoA* genes (Table 1) [13–15]. We identified 17 CRISPR spacer sequences but no integrated phages in the genome of strain Kam1 (Table 1).

**Table 1:** Genomic properties of the three “*Ca*. Methylacidiphilum” species for which a closed genome is available

	Kam1^a^	SolV^b^	V4^c^
Size (bp)	2,202,032	2,476,671	2,287,145
Contigs	1	1	1
GC %	40.34	41.48	45.48
RNAs	53	49	52
CDS	2119	2741	2473
16S	1	1	1
*pmoA*s	4	3	3
CRISPR spacers	17	23	20
Phages	0	0	0
Integrated plasmids	1	1	1
Transposable elements	12	80	37
Genomic islands	2	22	9
Locus tag prefix	Ga0255985_11^e^; kam1_^f^	Mfumv2_^g^; (Ga0069468_11)^e^	Minf_
IMG genome ID^d^	2,770,939,480	2,630,968,640	642,555,138
Genbank accession	CP037899	PRJEB6910	CP000975

^a^“*Ca*. Methylacidiphilum kamchatkense” Kam1, this study

^b^“*Ca*. Methylacidiphilum fumariolicum” SolV, [14]

^c^“*Ca*. Methylacidiphilum infernorum” V4, [13]

^d^Integrated Microbial Genomes and Microbiomes [17]

^e^Locus tag prefixes from Integrated Microbial Genomes and Microbiomes IMG/ER [17]

^f^Locus tag prefixes from National Center for Biotechnology Information (NCBI)

^g^Locus tag prefixes and annotation as given by Mohammadi and colleagues 2017 [16]

Until now, it has been unclear if strains SolV and Kam1 constitute two different species or not [3].

The average nucleotide identity (ANI) is a fast and easily reproducibly in-silico alternative to experimental DNA-DNA hybridization. An ANI value of 95% corresponds to the 70% species cutoff used for DNA-DNA hybridization [18]. Previously, a subsample of randomly chosen genes from SolV was used to calculate an ANI value of 73% for strains SolV and V4, clearly showing that they constitute two different species, “*Ca*. M. fumariolicum” and “*Ca*. M. infernorum”*,* respectively [3]. We used the closed genomes of strains Kam1, SolV and V4 to calculate the ANI between the three strains (Additional file 1: Table S1). The obtained ANI values between strains Kam1, SolV and V4 are all below 95%, therefore, they should be considered as type strains of three different species, “*Ca*. M. kamchatkense*”*, “*Ca*. M. fumariolicum” and “*Ca*. M. infernorum” respectively (Additional file 1: Table S1).

We then used the annotated genome for a detailed analysis, linking genomic data with metabolic and physiological traits reported in the literature. This analysis includes a comparison of the genomes of strains Kam1, SolV and V4. The result of which will be presented in detail in the discussion section.

## Discussion

### Mobile elements

Dot plot comparison of the three genomes revealed a very high degree of conservation of the genome architecture between strains Kam1 and SolV (Additional file 1: Figure S1-A), whereas several rearrangements and inversions of large genomic regions were identified when comparing strains Kam1 or SolV with V4 (Additional file 1: Figure S1-B-C).

The large number of rearrangements and the observation of several gaps in whole genome alignments of strains Kam1, SolV and V4, prompted us to investigate and compare the presence and abundance of mobile elements.

The presence of a ≈ 45 kbp putative integrative plasmid has been reported in the genome of strain V4 [13]. Whole genome alignments revealed that this putative integrative plasmid is present in both strain Kam1 and SolV, although some insertions or deletions has occured. The putative integrated plasmid is located in the same region of the genome in all three strains, showing that it was present in their last common ancestor. No prophages were found in any of the three genomes.

All three strains encode a class I, type III CRISPR-cas adaptive immune system, involved in protecting against phages and plasmids [19, 20]. The three genomes encode 17–23 CRISPR spacer sequences, all spacers are species specific but in a few cases, two identical copies are present within the same strain (Table 1, Additional file 2). The spacers and CRISPR associated genes are found in different regions in all three genomes. The Cas2 proteins from strains Kam1 and SolV are 81% identical and the gene order and content of the CRISPR regions are highly similar. In contrast, the two Cas2 proteins from strain V4 is only 14–16% identical to their Kam1/SolV counterparts and the entire CRISPR region differs from Kam1/SolV (Additional file 1: Figure S2). The *cas*1 gene from strain V4 is truncated; it is therefore likely that the CRISPR system of this strain is non-functional [13]. Whereas the CRISPR systems of strains Kam1 and SolV appears to be functional. The presence of different CRISPR systems in the genomes of strains Kam1/SolV and V4, suggests that either one or both has been acquired horizontally from different donors.

**Fig. 1:**
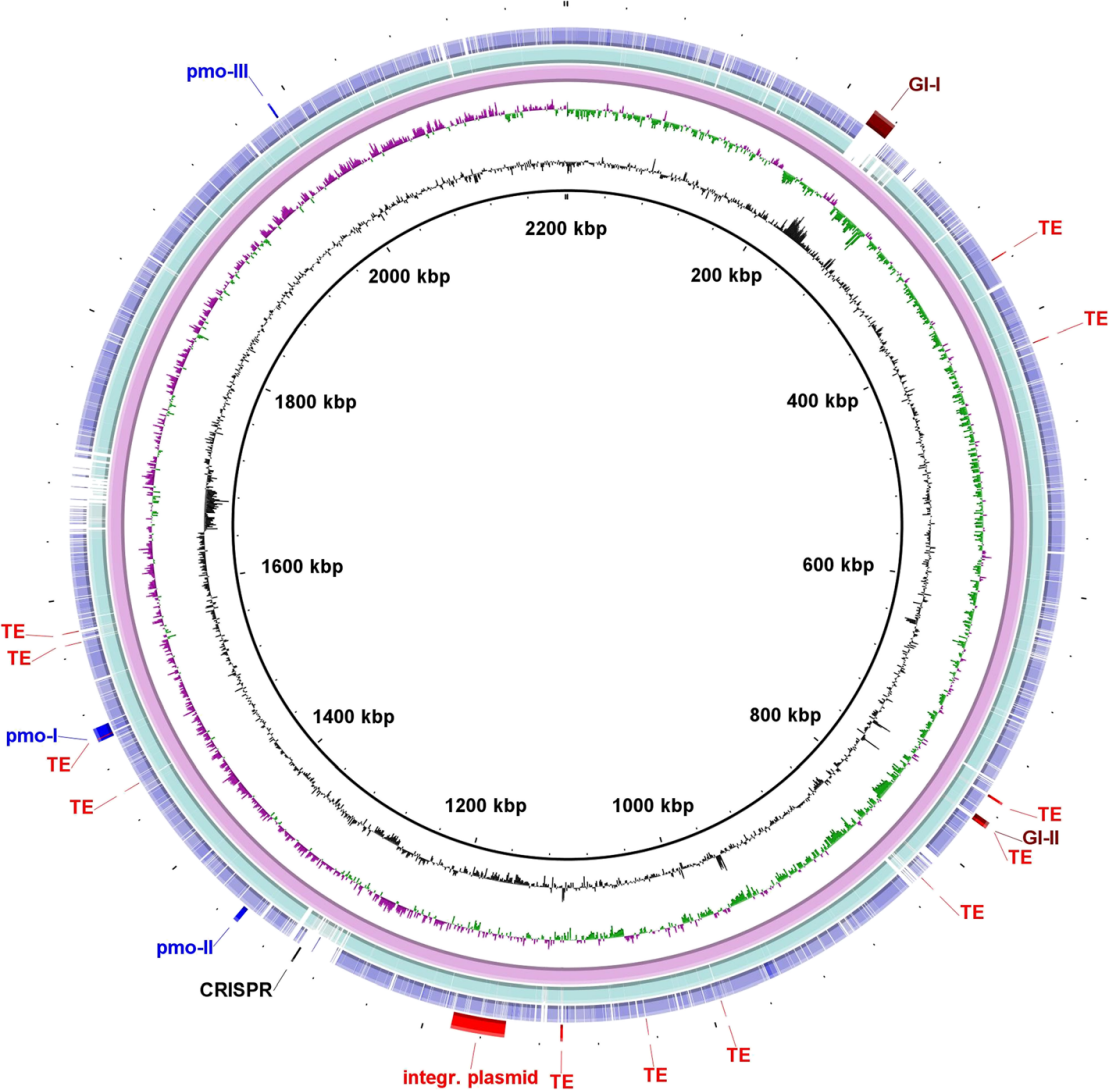
Circular representation of the genome of “*Ca*. Methylacidiphilum kamchatkense” Kam1. Rings from inside to outside: 1) GC content (black); 2) GC skew (−/+ purple/green); 3) strain Kam1; 4) strain SolV; 5) strain V4; 6) Selected genomic traits of strain Kam1, for exact genomic coordinates see (Additional file 2). GI: Genomic island; TE: Transposable element; Pmo: *pmo* cluster, see also Fig. 2

The genome of strain Kam1 encodes only two predicted genomic islands (GI)s with a size of 17 (GI-I) and 4,8 kb (GI-II) respectively, in contrast to the 22 and 9 predicted GIs with a total size of 205.5 kb and 111.4 kb found in the genomes of strains SolV and V4, respectively, (Table 1; Fig. 1, Additional file 1: Figures S3–S4). GI-I encodes 18 predicted genes, whereof 14 is without a predicted function; the majority of the genes with a predicted function encodes DNA modification systems. GI-II encodes four predicted genes, whereof two is without a predicted function (Additional file 1: Table S2). GI-I is not present in the genomes of strains SolV and V4, whereas GI-II is present in both strains Kam1, SolV, and partially in V4 (Fig. 1). Two out of five analyzed genes from GI-I, gave blastn hits to genomes of non-verrucomicrobial thermophiles and one to a plasmid from a mesophile, whereas the last two sequences, did not resemble any sequences in the databases. The four predicted genes from GI-II gave blastn hits to strains SolV and V4 and in two instances to other thermophilic bacteria (Additional file 1: Table S2). Similar observations were made for the GIs from strains SolV and V4 (data not shown). Since the majority of the genes found in GI-I and II is without a predicted function, it is not possible to say whether the presence of homologs in other unrelated thermophiles or thermoacidophiles means that they encode beneficial traits for survival in this niche or simply reflects horizontal gene transfer between organisms found in the same environment.

Transposons and other insertion sequences, from here on collectively referred to as transposable elements (TE), are small genomic elements capable of moving within or between genomes. Movement can be facilitated by genes such as transposases, encoded by the TE itself or by encoded sequences recognized by transposases encoded by other TEs, integration into f. ex. conjugative plasmids allows horizontal gene transfer of TEs (for review see [21]). The presence of TEs can have profound effects on bacterial genomes, by disrupting genes, altering gene expression or lead to deletions and rearrangements within the genome [21, 22].

We searched the genomes of strains Kam1, SolV and V4, available at the Joint genome institutes IMG/ER server for annotated transposases using the search term “transpos” [17]. We found four transposases annotated in the previously published draft genome of strain Kam1, whereas the closed genomes of strains Kam1, SolV and V4 encode 3, 48 and 23 annotated transposases, respectively [13–16].

In total we identified 12 TEs in the closed genome of strain Kam1 and a large number TEs in the genomes of strains SolV and V4 (Table 1 and Additional file 2). The TEs from all three “*Ca*. Methylacidiphilum” strains are generally located in regions with either no or low homology to the other two genomes, indicating that these are either species specific or in various stages of decay (Fig. 1 Additional file 1: Figures S3–S4).

**Fig. 2:**
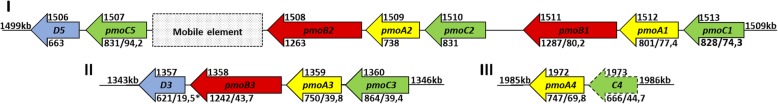
Organization of *pmo* cluster I-III from “*Ca*. Methylacidiphilum kamchatkense” Kam1. Numbers at the end of lines indicate location in the genome. Numbers on top of arrows indicate locus tags without prefix, (see Table 1 for prefixes), numbers under arrows indicate gene size in bp / % amino acid identity to the *pmo*_*2*_ homolog. * amino acid identity to PmoD_5_. Arrow surrounded by a dashed line indicates a truncated and thus likely non functional gene

It is likely that TEs have had a large impact on the evolution of “*Ca*. Methylachidiphilum”. Examples of this is the presence of TEs integrated into *pmo* cluster I, between *pmoB*_*2*_ and *pmoC*_*5*_ (Fig. 2), indications that movement of TEs have led to the loss of *pmoCA*_*4*_ from strains SolV and V4 and the loss of the type 1h NiFe hydrogenase from the genome of strain V4, as will be discussed below.

### Methane metabolism

Genome analysis confirmed the absence of genes encoding sMMO. The genome of Kam1 encodes three *pmoCAB* operons, a truncated *pmoCA* operon and a separate *pmoC* gene, as reported previously (Fig. 2) [3, 10]. The methane monooxygenases from “*Ca*. Methylacidiphilum spp.” and other verrucomicrobia constitute a distinct group, clearly separated from their proteobacterial counterparts. The *pmoA* gene is present in all known aerobic methanotrophs, and is commonly used as marker gene, for detection of potential for methanotrophy in environmental samples. The primers used for detection of *pmoA* from proteobacteria, do not amplify *pmoA* genes from verrucomicrobia nor from the intra-aerobic members of the candidate phylum NC10 under standard PCR conditions. This may have led to an underestimation of the abundance and diversity of methanotrophs in some environments [3, 23].

Recently, a primer pair amplifying the intergenic region between *pmoA* and *pmoB* has been reported to target both *pmoCA* from proteobacteria and *pmoCA*_1–2_, but not *pmoCA*_*3*_, from strain SolV [24].

The genome of strain Kam1 encodes three *pmo* gene clusters (I-III, Fig. 2). I) Organized as one large cluster encoding two *pmoCAB* operons in tandem and a downstream orphan *pmoC.* II) Encoding a single *pmoCAB* operon and finally III) encoding a *pmoCA* operon, unique for strain Kam1, where *pmoC*_*4*_ are N-terminally truncated (Fig. 2, Table 2. Additional file 1: Table S3) [10]. Changing a single base at position 1,986,490, would change an ATT to an ATG start codon, on the reverse strand, restoring a full-length *pmoC*_*4*_ with a size of 831 bp (data not shown).

**Table 2:** Presence (√), absence (−--) or number of genes or groups of genes encoding selected metabolic traits

	Kam1	SolV	V4
*pmo* clusters	3	2	2
Uptake hydrogenase, NiFe, type 1h	√	√	–
Uptake hydrogenase, NiFe, type 1d	√	√	√
Solouble hydrogenase, NiFe, type 3b	√	√	√
Calvin Benson Bassham cycle	√	√	√
MoFe-Nitrogenase	√	√	√
Alternative MQ synt. Pathway	√	√	√
Resp. Complex I, 14 subunits	√	√	√
Resp. Complex II	√	√	√
Complex III, bc1	–	–	–
Alternative complex III	√	√	√
Resp. Complex IV CBB3 type	√	√	√
Resp complex V, ATP synthase	2	2	2
Ech hydrogenase related (Ehr) complex	√	√	√
Heavy metal efflux pumps	8	8	9

Strain designations are given on top of columns. For full species, names see Table 1

The majority of the organisms encoding *pmoCAB,* also encodes a membrane protein PmoD [25, 26]. The physiological role of PmoD, and the closely related AmoD, is still not understood. Their encoding genes are usually found as part of *pmo/amoCAB* operons, but homologs next to Cu resistance genes have also been reported [25]. A role as a copper chaperone has been proposed [25, 27]. This speculation is supported by expression data from *Methylococcus capsulatus* Bath grown in the presence or absence of copper. The expression of a *pmoD* homologue, MCA2130, located next to a multicopper oxidase family protein was down-regulated in the absence of copper, whereas another *pmoD* homologue, MCA2170b, located next to a *copC* gene, was upregulated [28]. This is consistent with the speculation that CopC has a role in copper uptake, in organisms possessing the copper dependent particulate methane monooxygease [29]. A recent study demonstrated that PmoD is a membrane bound copper binding protein. Furthermore, knocking out *pmoD* in *Methylosinus trichosporium* OB3b resulted in inability to grow using the Copper dependent pMMO, whereas growth using the copper independent sMMO was unaffected [26].

Careful inspection of the genome revealed the presence of a *pmoD* homologue located directly downstream of *pmoC*_*5*_. A blastP search against the entire genome using the sequence of this *PmoD*_*5*_ as a query revealed a total of four *pmoD* homologs in the genome of strain Kam1. Two of these are part of *pmo* clusters I or II, one is located next to a gene annotated as multicopper oxidase, and one is located in close proximity to a CRISPR region (Fig. 2. Additional file 1: Table S4). All four *pmoD* homologs encode a single C terminal transmembrane helix, and two out of four encode a signal sequence (Additional file 1: Table S4). We found homologs of all four *pmoD*s in both strains SolV and V4, although they appear to be slightly less conserved between the three strains than the other *pmo* genes (Additional file 1: Tables S5-A-D).

**Fig. 3:**
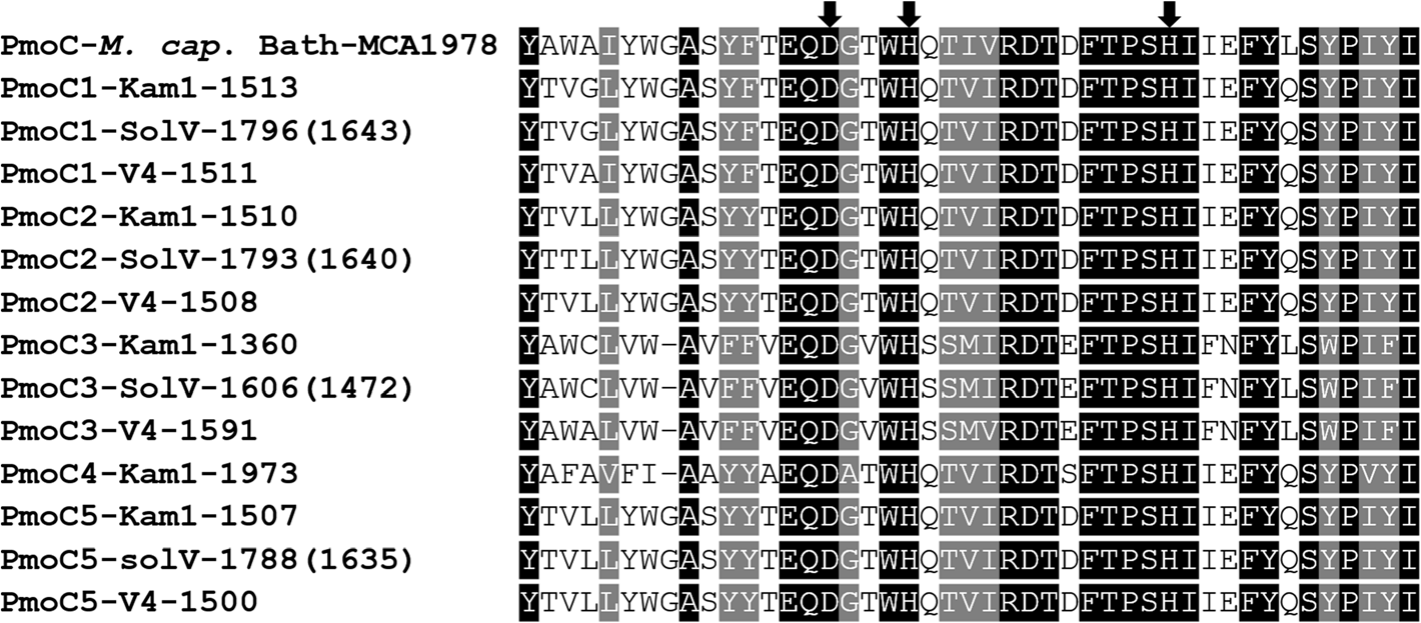
Alignment of the PmoCs from strains Kam1, SolV and V4 with PmoC from *Methylococcus capsulatus* strain Bath. PmoC number and strain designations are given followed by locus tags without prefix, (see Table 1 for prefixes). The conserved copper binding motif (DxxxH(x_12_)H) is indicated with black arrows. For strain SolV locus tags without or between brackets refers to the annotation following Mohammadi and colleagues 2017 [16] or Integrated Microbial genomes and Microbiomes IMG/ER [17] respectively, see also (Table 1)

The verrucomicrobial *pmoB* genes do not encode the conserved Copper binding sites found in all other PmoBs [3, 30]. Untill recently it was believed that the active site of pMMO is located in the PmoB subunit [31]. However, the authors of a recent paper found that the active site isn’t located in PmoB. In the same paper they found evidence that a predicted zinc/copper binding site (DxxxH(x_12_)H) in PmoC, binds copper, the authors speculated that this may be the active site [32]. Interestingly the (DxxxH(x_12_)H) motif is conserved in all PmoCs from strains Kam1, SolV and V4 (Fig. 3).

The conservation of the copper binding (DxxxH(x_12_)H) motif in all PmoCs; the identification of *pmoD* homologs as part of the *pmo* gene clusters in all three “*Ca*. Methylacidiphilum spp.” strains and the observation that growth of strain Kam1 ceased after two transfers in copper free medium. Strongly suggests that the pMMOs of strain Kam1 and likely other “*Ca.* Methylacidiphilum” strains are copper dependent.

The presence of multiple copies of the *pmo* genes in strain Kam1, and other methylacidiphila, may be the result of gene duplications. In support of this speculation, the *pmo* genes located in close proximity of each other are more similar than homologs located more distantly in the genome. Exemplified by the *pmoC*s from strain Kam1 where the co-located *pmoC*_*1*_, *C*_*2*_ and *C*_*5*_ are 74–94% identical to each other but only 39–59% identical to *pmoC*_*3*_ and *C*_*4*_, that are located elsewhere in the genome (Additional file 1: Table S5-C).

It has been shown that *pmoCAB*_*1–3*_ from strains Kam1, SolV and V4 are highly conserved between strains and under intense purifying selection, suggesting that they have evolved to have distinct roles under different conditions [3, 10]. Furthermore, *pmoCAB*_*2*_ is the highest expressed methane monooxygenase under non-limiting growth conditions in both strains Kam1 and SolV [10, 33]. Whereas transcriptomics analysis of strain SolV showed a shift from *pmoCAB*_*2*_ to *pmoCAB*_*1*_ under oxygen limiting growth conditions [33].

Genome alignments of strains Kam1, SolV and V4 showed that *pmo* cluster I is located in the same part of the genome in all three strains, although a number of transposable elements are found within and surrounding this cluster (Additional file 1: Figure S5). *Pmo* cluster II, encoding *pmoCABD*_*3*_, is also located in the same part of the genomes of strains Kam1 and SolV, but is integrated in another part of the genome of strain V4, showing that the whole region has moved within the genome. Full genome alignment revealed large numbers of genomic rearrangements on both sides of *pmoCABD*_*3*_ (Additional file 1: Figure S6). The genome of strain Kam1 encodes *pmo* cluster III encoding *pmoCA*_*4*_, that are not found in the genomes of strains SolV and V4 [3].

Interestingly we found that *pmoCA*_*4*_, has been replaced by transposable elements in strains SolV and V4. In strain SolV we found fragments of *pmoC*_*4*_ at the 5′ and 3′ end of the inserted transposable element, showing that *pmo* cluster III was present in the last common ancestor of strain Kam1 and SolV (Additional file 1: Figure S7).

**Fig. 4:**
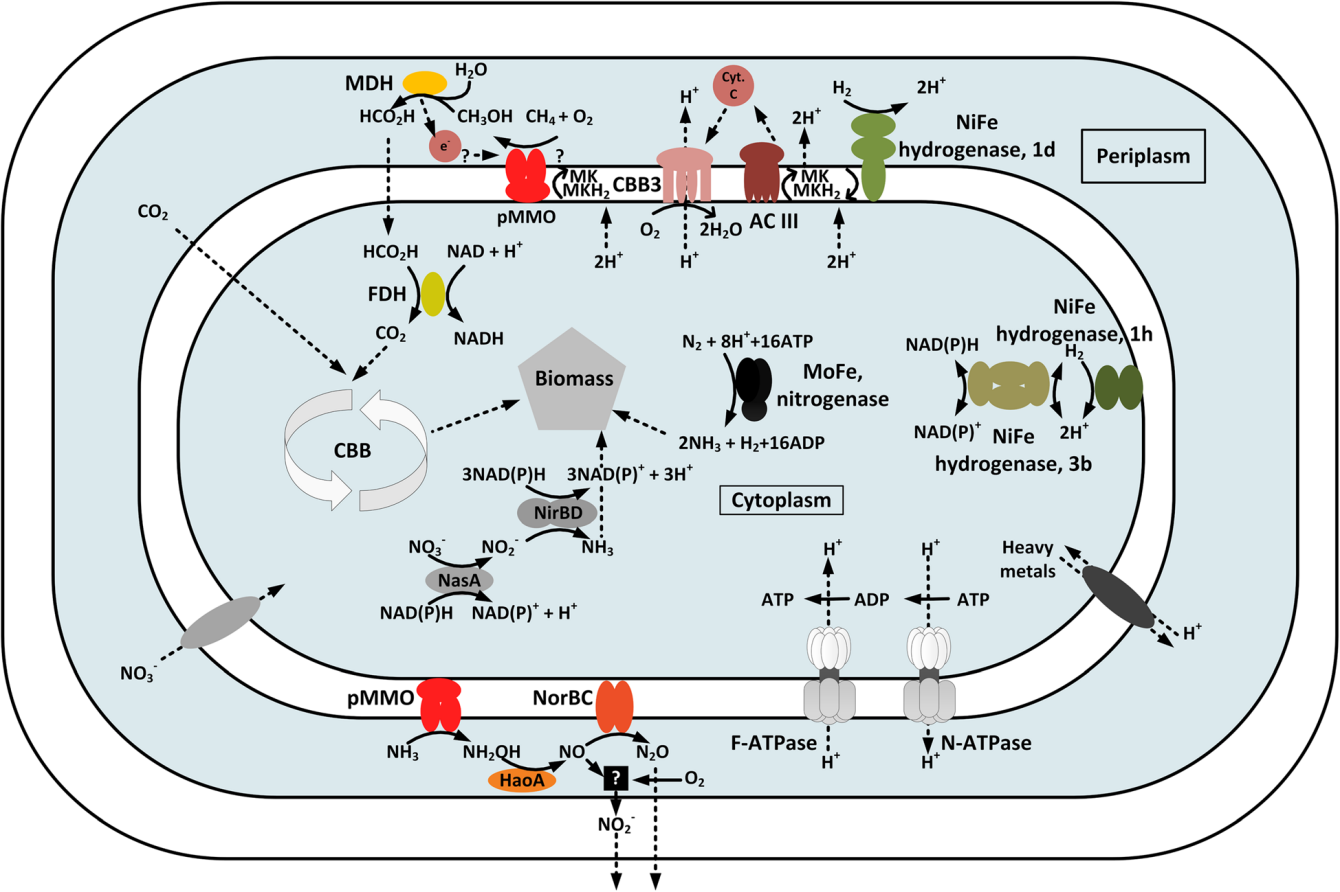
Schematic representation of “*Ca*. Methylacidiphilum kamchatkense” Kam1 showing selected metabolic traits. MDH: methanol dehydrogenase; pMMO: particulate methane monooxygenase; CBB3: cbb3 type cytochrome oxidase; AC III: alternative complex III; MK: menaquinone; FDH: formate dehydrogenase; CBB: Calvin Benson Bassham cycle; NasA: assimilatory nitrate reductase; nirBD: nitrite reductase; HaoA: hydroxylamine oxidase; NorBC: nitric oxide reductase; Black box with a question mark: NO oxidation, either nonenzymatic or catalyzed by a yet unidentified NO oxidase

In methanotrophs the methanol produced by the pMMOs oxidation of methane is subsequently oxidized by a methanol dehydrogenase (MDH). The genome of Kam1 does not encode a calcium dependent MDH of the MxaF-type, instead it encodes a XoxF-type, lanthanide dependent MDH. The encoding gene is part of a small operon *xoxFJG:J* that is also found in the genomes of strains SolV and V4 (Additional file 1: Table S3) [13, 14]. In brief, *xoxF,* encodes the lanthanide dependent MDH. *xoxJ,* encodes a protein speculated to have a role in binding the MDH to pMMO. *xoxG:J*, encodes a XoxG and XoxJ fusion protein. The XoxG part is a cytochrome c, that acts as electron acceptor for the MDH, the XoxJ part is truncated compared to the non-fused XoxJ, and has been speculated to have a role in binding XoxG:J to the MDH [12, 34]. Both MDHs of the MxaF and XoxF-type requires co-factor PQQ [12]. The genomes of all three strains encodes genes, *pqqABCDEFG*, for biosynthesis of PQQ, these are organized as a *pqqABCDE* cluster with *pqqF* and *pqqG* located elsewhere in the genomes (Additional file 1: Table S3). MDHs catalyzes the oxidation of methanol to formaldehyde, that then can be further oxidized to CO_2_ via formate or assimilated into biomass. Formaldehyde is not assimilated by methanotrophic verrucomicrobia, as will be discussed in the “biomass generation” section [8, 11]. It has been demonstrated that MDH from SolV oxidizes methanol and formaldehyde with the same maximum rate. Leading to the speculation that methanol is oxidized directly to formate [12]. The formate is then further oxidized to CO_2_ by a NAD dependent formate dehydrogenase (Fig. 4) (Additional file 1: Table S3). In line with this speculation, the genomes of strains Kam1, SolV and V4 do not encode the tetrahydrofolate, tetrahydromethanopterin or the glutathione pathway for formaldehyde oxidation [13, 14, 35].

### Biomass generation

Traditionally methanotrophs have been divided into two groups based on how they assimilate formaldehyde. Group one uses the RuMP pathway, whereas group two uses the Serine pathway [2]. Our analysis showed that strain Kam1, like strains SolV and V4, encodes incomplete RuMP and serine pathways. Genes encoding hexulose-6-phosphate synthase and 6-phospho 3-hexuloisomerase are lacking from the RuMP pathway, and genes encoding Malyl coenzyme A lyase and Glycerate kinase from the serine pathway. Instead it, like strains SolV and V4, encodes a full Calvin Benson Bassham pathway for CO_2_ fixation (Table 2, Additional file 1: Table S6) [3, 13, 14]. Khadem and colleagues demonstrated that strain SolV obtains carbon by fixing CO_2_ via the CBB pathway rather than fixing formaldehyde as conventional methanotrophs [11]. Similar observations has been done for three mesophilic methanotrophic strains from the closely related *methylacidimicrobium* genus [8]. Most likely this is also the case for strains Kam1 and V4, and possibly a common trait of methanotrophic verrucomicrobia.

We found large numbers of genes associated with glycogen metabolism, in the genome of strain Kam1, all with homologs in the genomes of strains SolV and V4 (Additional file 1: Table S7). Strain SolV has been shown to use glycogen stored in cytoplasmic glycogen vesicles as carbon and energy storage [36]. Similar vesicular structures were observed in strains Kam1 and V4, it is thus plausible that all three strains use glycogen as carbon and energy storage [3, 7]. Some methanotrophs use poly-β-hydroxybutyrate as carbon and energy storage [37]. We did not identify key genes, *phbABC,* from the poly-β-hydroxybutyrate synthesis pathway in the genome of strain Kam1. These genes are also not present in the genomes of strains SolV or V4 [13, 36].

### Hydrogenases

Recently it was demonstrated that strain SolV is capable of autotrophic growth on H_2_, O_2_ and CO_2_ [16]. Similarly, it has been shown that “*Ca*. Methylacidiphilum sp.” strain RTK17.1, that is closely related to strain V4, employs a mixotrophic lifestyle, oxidizing methane and hydrogen simultaneously [9].

The genome of strain Kam1 encodes three NiFe hydrogenases, classified as type 1d, 1h and 3b using the hydDB classification tool [38]. The type 1d and 3b hydrogenases are also present in strains SolV and V4, whereas the type 1h is only found in the closely related strains Kam1 and SolV (Table 2, Additional file 1: Table S8) [13, 14].

The 1d hydrogenase is an oxygen tolerant, membrane attached, uptake hydrogenase encoded by *hyaABC*. In brief, *hyaB* encodes the catalytic large subunit, *hyaA* encodes an FeS protein with a Tat signal sequence, the small subunit, and *hyaC* encodes a membrane bound cytochrome B. Electrons are transferred from HyaB via HyaB to HyaC and further to the quinone pool [39]. We suggest that the type 1d hydrogenase is anchored in the membrane and facing the periplasm as depicted in Fig. 4.

The type 3b hydrogenase is an oxygen tolerant cytoplasmic NADP dependent hydrogenase encoded by *hyhBGSL*. In brief, *hyhSL* encodes the small and large subunits of the hydrogenase, respectively. The *hyhL* gene contains a frameshift mutation, that is not present in the draft genome of strain Kam1, and thus may be a sequencing or assembly error [15]. The *hyhBG* genes encode the electron transfer protein and the catalytic subunit respectively of the predicted NADPH dehydrogenase. It should be noted that the only characterized type 3b hydrogenase comes from the archeon *Pyrococcus furiosus*; it has not yet been tested if the bacterial type 3b hydrogenases are NADP dependent [40].

The type 1h hydrogenase is an oxygen tolerant high affinity uptake hydrogenase encoded by *hhySL* coding for the large and small subunit respectively [41].

The gene cluster encoding a type 1h/5 type hydrogenase in *Mycobacterium smegmatis* strain MC^2^ 155 encodes an FeS protein, HhyE, speculated to act as an electron transfer protein linking the hydrogenase to the cells electron transport chain [42]. We did not find a *hhyE* gene in close proximity to *hhySL* in strains Kam1, SolV or V4. A blastP search, using MSMEG_2718 coding for HhyE in *Mycobacterium smegmatis* strain MC^2^ 155, against the genomes of strains Kam1, SolV and V4 gave no hits, indicating that they do not possess *hhyE* genes located elsewhere in the genomes.

The products of six genes, *hypABCDEF,* are necessary for maturation and incorporation of metal cofactors in the active site of NiFe hydrogenases [43]. We found the full *hypABCDEF* gene set in the genomes of strains Kam1, SolV and V4. These are organized as a *hypBCDEF* gene cluster and a *hypA* located elsewhere in the genome (Additional file 1: Table S8).

In strains, Kam1 and SolV, *hypBCDEF* are co-located with the type 1h hydrogenase, *hhySL,* and *hypA* is located approximately 69 kbp downstream of these. Full genome alignments showed that the genomic region surrounding *hypABCDEF* is conserved between strains Kam1 and SolV. Aligning the genomes of Strains Kam1 and V4 revealed several rearrangements in the regions harboring *hypBCDEF* and *hypA*. The *hhySLhypBCDEF* genes are located on an approximately 26 kbp genomic region that is conserved between strains Kam1 and SolV. This region is absent from the genome of strain V4, with the exception of *hypBCDEF* that is found in another part of the genome and encoded on the negative strand (Additional file 1: Figure S8). Similar observations were done for the genomic region encoding *hypA* (Data not shown). Based on these observations, we speculate that the type 1h hydrogenase was present in the last common ancestor of strains Kam1, SolV and V4 and then subsequently lost from strain V4 due to genomic rearrangements.

### Nitrogen metabolism

Strain Kam1 is able to use N_2_, nitrate and ammonium as nitrogen source [7]. The genomes of strains Kam1, SolV and V4 encode *nifHDKENB*, the minimum gene set required for nitrogen fixation (Table 2, Additional file 1: Table S9) [13, 44, 45]. In brief, *nifHDK* encodes the MoFe nitrogenase, and *nifENB* are needed for assembly and insertion of the FeMoco co-factor [44]. The nitrogenase encoding genes are part of a 29.5 kbp gene cluster, conserved between all three strains. In addition to the *nif* genes, this gene cluster also encodes *fixABCX* and several genes predicted to be involved in regulation and maturation of the nitrogenase. The product of the *fix* genes couples oxidation of NADH with the simultaneous reduction of high potential quinones and low potential flavodoxins via electron bifurcation. It is believed that this process generates the low potential reductants needed for nitrogen fixation [46]. For strains Kam1 and V4 indirect proof of nitrogen fixation stems from growth in medium without a nitrogen source, whereas nitrogen fixation has been directly demonstrated for strain SolV [3, 7, 47].

Nitrate assimilation to biomass, requires that nitrate is reduced first to nitrite and then further to ammonium, a process requiring a total of eight electrons [48] and references therein.

**Fig. 5:**
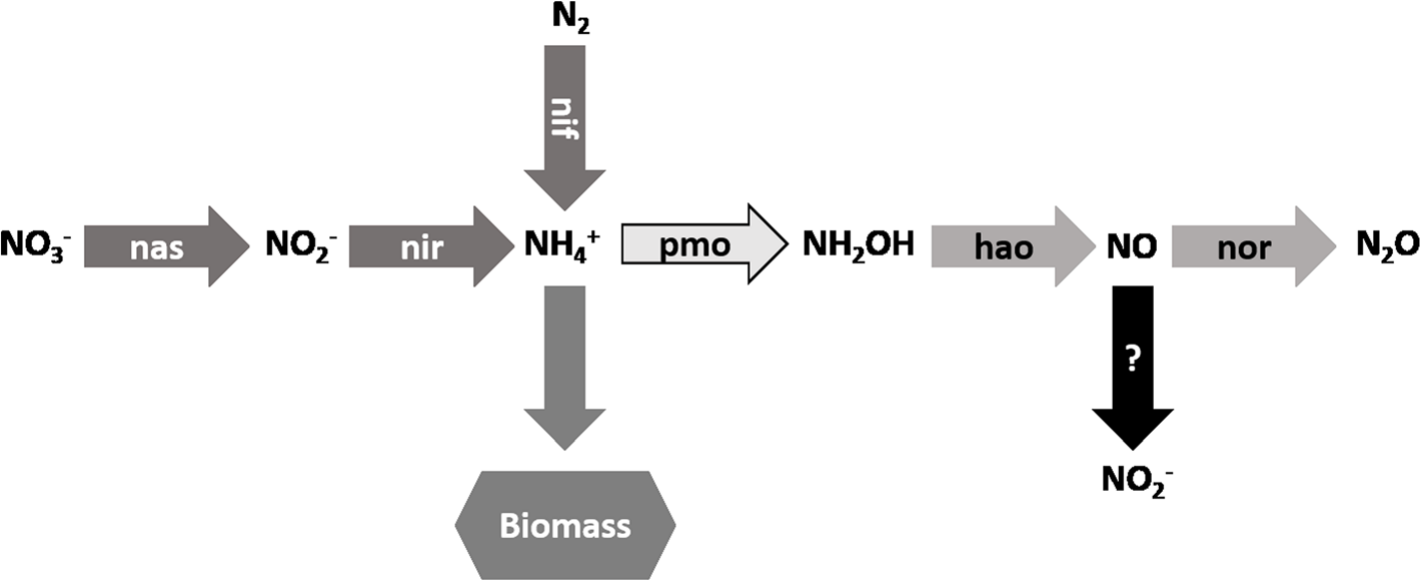
Overview of the nitrogen metabolism of “*Ca*. Methylacidiphilum kamchatkense” Kam1. Letters inside arrows refers to the enzymes catalyzing the reaction, see (Additional file 1: Table S9) for locus tags. Dark gray arrows: nitrogen assimilation; light gray arrows: detoxification; white arrow: fortuitous reaction. Nas: assimilatory nitrate reductase; Nir: Nitrite reductase; Nif: nitrogenase; pmo: particulate methane monooxygenase; Hao: hydroxylamine oxidase; Nor: nitric oxide reductase; Black arrow with a question mark: NO oxidation, either nonenzymatic or catalyzed by a yet unidentified NO oxidase

The genomes of strains Kam1, SolV and V4 encode a nitrate reductase, NasA, and a nitrite reductase nirBD, both predicted to be localized in the cytoplasm (Figs. 4 and 5, Additional file 1: Table S9). In brief, NasA catalyzes the reduction of nitrate to nitrite and NirBD the reduction of nitrite to ammonia.

The *nasA* and *nirBD* are found in a nitrogen assimilation gene cluster together with predicted ammonium, nitrate and nitrite transporters. This gene cluster is conserved between all three strains (Additional file 1: Table S9).

The methane monooxygenase, pMMO, and the ammonia monooxygenase, AMO, are highly similar homologous enzymes. In agreement with this, AMO is capable of methane oxidation and pMMO of oxidizing ammonium to hydroxylamine [30, 49]. Hydroxylamine is highly toxic, and must therefore be removed from the cells. Hydroxylamine is oxidized by hydroxylamine oxidoreductase, a process that in ammonia oxidizing bacteria is coupled to energy conservation, but in methanotrophs serves as part of a detoxification mechanism [50]. The genomes of all three strains encode a hydroxylamine oxidoreductase, HaoAB, and a nitric oxide reductase NorBC (Figs. 4 and 5, Additional file 1: Table S9). The traditional view has been that HaoAB catalyzes the four-electron oxidation of hydroxylamine to nitrite. However it has recently been shown that HaoAB catalyzes a three electron oxidation of hydroxylamine to nitric oxide [51, 52]. It is therefore likely that they dispose of toxic hydroxylamine by first oxidizing it to nitric oxide that is then reduced to nitrous oxide that diffuses out of the cell (Figs. 4 and 5). However, ammonia oxidation by both methanotrophs and ammonia oxidizing bacteria leads to formation of nitrite (Figs. 4 and 5). The source of the nitrite is still under debate. It has been shown that NO is oxidized by O_2_ in a nonenzymatic manner leading to formation of nitrite. However there is evidence that ammonia and methane oxidizing bacteria encodes a hitherto unidentified NO oxidase [51, 52]. The nitrite reductase NirK catalyzing the reduction of nitrite to NO, has been proposed as a possible candidate for this unidentified NO oxidase, by operating in the reverse direction [51]. Interestingly, strain SolV encodes two putative nitrite reductases (Mfumv2_1120 and 1973). We found a homolog of Mfumv2_1120 in the genome of strain V4 whereas we did not find any nitrite reductases in the genome of strain Kam1 (Additional file 1: Table S9). It has previously been suggested that Mfumv2_1973 encodes a nitrite reductase, NirK, responsible for nitrite reduction in strain SolV, although transcription analysis showed transcription of both Mfumv2_1120 and 1973 [53]. Whole Genome alignments revealed that the genomic regions directly flanking the genes encoding putative nitrite reductases, in strain SolV, are conserved between all three strains (Additional file 1: Figure S8). We performed a blastn search against the NCBI database (17-12-2018) using Mfumv2_1020 and 1973 from strain SolV as query. The best hits for Mfumv2_1120 and 1973 respectively were to sequences from *Nitrosomonas eutropha* (83% coverage, 67% identity) and *Oligotropha carboxidovorans* (43% coverage, 67% identity) and other proteobacteria (Data not shown), indicating that these genes may have been acquired by horizontal gene transfer.

### Quinones and respiration complex I to V

Quinones, are freely diffusible lipophilic electron carriers acting as electron carriers in the membrane [54]. The genome of strain Kam1 does not encode a ubiquinone nor the classical, *menFDHCEBIAG*, Menaquininone (MK) synthesis pathway [55–57]. Instead, we found genes from the alternative, futalosine, MK synthesis pathway in the genomes of all three strains (Table 2, Additional file 1: Table S10). We were unable to identify a *mqnB* gene, the product of which is predicted to catalyze the conversion of futalosine or aminodeoxyfutalosine to dehypoxanthinyl futalosine. However, variations of the futalosine pathway are known to exist, it is thus likely that the methylacidiphila encode a yet uncharacterized variant [58, 59].

Genes encoding respiration complex I to V has been identified in “*Ca*. Methylacidphilum sp.” strain RTK17.1 [9]. We found homologs of these in the genomes of strains Kam1, SolV and V4, indicating that they are conserved among methylacidiphila (Table 2, Additional file 1: Table S10). NADH:ubiquinone oxidoreductase (complex I) and succinate dehydrogenase (complex II) represents two entry points of electrons into the quinone pool. The genome of all three strains encodes the classical 14 subunits complex I, consisting of NuoA-N. It is a membrane complex transferring two electrons from NADH to quinones in the membrane while translocating four protons across the cell membrane, thereby creating a proton gradient [60] and references therein. Complex II, is a cytoplasmic orientated membrane complex encoded by *sdhABC*. Complex II links the Tricarboxylic acid cycle (TCA) with the respiration chain by transferring two electrons from succinate to the quinone pool, while oxidizing succinate to fumarate [61]. The genome does not encode a complex III, also called BC_1_ complex, instead it encodes a structurally unrelated but functionally similar complex, named alternative complex III (ACIII). This complex transfers electrons from reduced quinones via a cytochrome c to complex IV, thereby regenerating the quinone pool. It has been proposed that ACIII, also translocates protons across the cell membrane, but this still needs to be experimentally verified [62, 63]. The genes encoding the ACIII complex is co-located with genes encoding a cbb3 type cytochrome c oxidase (complex IV) (Additional file 1: Table S10). This type of cytochrome c oxidases have very high affinity for oxygen and are often associated with growth under low oxygen tension [64, 65]. This is in line with the observed high oxygen affinity of strain SolV [6], and our observation that strain Kam1 grows optimally at low oxygen concentrations.

The ATP-synthase, complex V, represents the final step in the electron transport chain where the proton gradient generated by the previous steps are consumed to produce ATP.

Previously the presence of two operons encoding different H^+^ translocating F-ATPases were reported from the genome of strain V4 [13]. We found homologs of these operons in the genome of both strains Kam1 and SolV [14]. One is most similar to the F-ATPase found in other verrucomicrobia, whereas the other resembles ATPases found in gamma-proteobacteria [13]. The gene order and content differs between the two operons (Table 2, Additional file 1: Table S10, and Figure S10). The operons encoding the verrucomicrobial and gamma-proteobacterial ATPases is organized as *atpBEFHAGDC* or *atpDCQBEF:HAG* respectively (Additional file 1: Figure S10). The organization of the latter resembles that of the Na^+^ translocating N-ATPases, *atpDCQRBEFAG*, first described by Dibrova and colleagues [66]. N-ATPases are always found in addition to a standard F-ATPase, and are thought to have a role in maintaining cell homeostasis [66]. There are two notable differences between these and the N-ATPase like operon found in strains Kam1, SolV and V4; Firstly, the absence of *atpR*, which is speculated to interact with the c-subunit [66]. Secondly, the *atpE* gene, encoding the c-subunit does not encode the Na^+^ binding domain ESTxxY. Recently an H^+^ translocating N-ATPase from the pathogen *Burkholderia pseudomallei* was characterized. It was suggested that this N-ATPase acts as a highly efficient H^+^ pump, enabling the cells to survive the low pH inside phagosomes [67]. It is tempting to speculate that in strains Kam1, SolV and V4, the F-ATPase are used for synthesis of ATP, whereas the N-ATPase like ATPase has a role in maintaining cell pH-homeostasis, similarly to what has been suggested for other N-ATPases [66, 67].

### Resistance to low pH and heavy metals

Acidophiles often have to cope with the dual stress of low pH and high loads of metals, since metals are more soluble at low pH [68]. Bacteria can protect themselves against low pH by passive and active means; the exact mechanism of microbial acid resistance is still not fully understood. Passive mechanisms includes adaptations of the cell membrane making it less permeable for protons and inversion of the membrane potential. Active mechanisms involves removal of protons from the cytoplasm by sequestering and translocation across the inner membrane reviewed in [69, 70]. Hou and colleagues identified a number of genes with a predicted function in acid resistance in the genome of strain V4 [13]. We identified homologs of these and other genes that may contribute to acid resistance in the genomes of both strains Kam1 and SolV (Additional file 1: Table S11).

The genomes of strains Kam1, SolV and V4 encodes a range of traits that may have a role in maintaining pH homeostasis of the cell, in addition to the proton translocation steps linked to the activity of respiration complex I to V discussed above.

We found genes with high homology to *gadBC* encoding the glutamic acid dependent acid resistance (GDAR) system in *Escherichia coli*. In brief, *gadB* encodes a glutamate decarboxylase catalyzing the proton consuming conversion of glutamate to 4-aminobutanoate and *gadC* encodes a glutamate/4-aminobutanoate antiporter (for review see [70]). The genomes also encode genes that may have a function analogous to the arginine-dependent acid resistance (ADAR) system in *E. coli*, as suggested for strain V4 [13]. *E. coli* encodes two arginine decarboxylases, a biosynthetic, *speA*, and an acid induced *adiA*. The ADAR system consists of *adiA* and an arginine/agmatine antiporter encoded by *adiC*. The genomes of all three strains encode homologs of *speA* and *adiC*, but not *adiA* (Additional file 1: Table S11).

The genome encodes three genes annotated as either Na-proline symporter or Na/H+ antiporters in addition to an energy conserving hydrogenase related complex (*ehrABCDLS*) and a gene, *ovp1*, encoding a H^+^-PPase (Additional file 1: Table S11) [71–73]. All of which may have a role in maintaining pH homeostasis of the cell.

Strain V4 has been reported to encode a mercury resistance system consisting or *merRAT:P* [13]. The functions are in brief; MerR: transcriptional regulator; MerA: mercury reductase and MerT:P: a fusion protein (Transporter classification database number 1.A.72.3) [74]; MerP is involved in binding mercury in the periplasm before transferring it to MerT that transports mercury across the inner membrane [75]. The *mer* genes are also found in strains Kam1 and SolV, but in these strains the *merT:P* and the *merRA* genes are located at separate places in the genomes (Additional file 1: Table S12).

The genome of all three strains encodes an arsenate resistance gene cluster *arsCR-acr3* and an additional orphan *arsC* homologue located elsewhere in the genomes. The functions are in brief; *arsC*, arsenate reductase; *arsR*, transcriptional regulator and *acr3*, AS (III) efflux pump [76, 77]. Our tests showed that strain Kam1 grows well in the presence of 1 mM As (III) or As(V), but not in the presence of 5 mM of either (Data not shown). Hou and colleagues identified 10 gene clusters encoding TolC, outer membrane proteins and/or AcrA, linking outer and inner membrane channels in addition to genes encoding a putative tellurium and a silver efflux pump (COGs 1538, 0845, 0861 and 3696, respectively) [13, 78]. Our analysis showed that all but one of these gene clusters are conserved between all three strains (Additional file 1: Table S12). We also identified two cation transport ATPases (COG 2217), these are also conserved between all three strains (Additional file 1: Table S12) [78].

### Conclusions

We here present the closed genome of “*Ca*. Methylacidiphilum kamchatkense” strain Kam1. We used genome analysis to show that strains Kam1 and SolV belongs to two different but closely related species. Our analysis revealed large differences in the numbers of TEs between the three “*Ca*. Methylacidiphilum” spp. We also found evidence that much of the differences between the three strains can be explained by the action of TEs, exemplified with the loss of a type 1h hydrogenase from the genome strain V4 caused by genomic rearrangements. Most of the genes encoding metabolic traits discussed in the present contribution are conserved among all three strains, although they may be found at different places in the genomes due to genomic rearrangements. Finally we present evidence that, *pmoCA*_*4*_ that is unique for strain Kam1, has been present and subsequently lost from the genomes of strains SolV and V4. The availability of three closed genomes have allowed us to do comparative analysis to gain a deeper understanding of the evolution and metabolism of this novel genus. Future studies should focus on linking genome and wet lab experimentation to improve our understanding of their metabolism. It is likely that more genomes from this genus will be available in the future, which will allow further comparative genomics analysis to elucidate even more details of evolution and adaptations to specific niches.

## Methods

### Cultivation, DNA extraction and sequencing

Strain Kam1 were originally isolated from an acid hot spring in Kamchatka, Russia by members of our research group at the University of Bergen, Norway as previously described [7]. The culture used in the present contribution originates from our in house culture collection. It has thus, not been necessary to obtain permission from any third parties, to use this strain in the present study. Strain Kam1 was cultivated at 55 °C and pH 3.5 with methane as the sole carbon and energy source as described in [10].

Cells were harvested by centrifugation of an early stationary phase culture, before DNA was extracted using the cetyltrimethylammonium bromide method [79].

The genome was sequenced using PacBio technology and assembled with HGAP3 [80], resulting in a single contig of 2,221,048 bp with an average coverage of 489. Genome sequencing and assembly were done by GATC, Konstanz, Germany. Overlapping ends of the linear contig were manually trimmed as recommended by Chin and colleagues [80], resulting in a final genome size of 2,202,032 bp.

### Genome annotation and analysis

Genome annotation and analysis were done using the Integrated Microbial Genomes and Microbiomes IMG/ER platform [17]. Signal sequences were identified with signalP 5.0 [81].

Jspecies were used to calculate Average Nucleotide Identities, [82]. Artemis V16 and Artemis comparison tool V13, were used for visualizing and comparing genomes [83, 84]. Circular genome comparison figures were generated with Blast Ring Image Generator (BRIG) [85]. Dotplots were calculated with Gepard [86]. Protein similarity and identity matrixes were generated with Matrix Global Alignment tool (MATGAT) with default settings [87].

We searched all three genomes for the presence of partial and decayed transposases using the nucleotide sequences of genes annotated as transposases from all three strains as a query for a discontiguous megablast E-10. Insertion sequences were identified with ISsaga www-is.biotoul.fr, predicted sequences flagged as probably false positives were manually curated [88]. Finally, data on annotated transposases, partial transposases and insertion sequences were combined and are for simplicity collectively referred to as transposable elements.

Hydrogenases were classified with HydDB [38]. Local genome searches and alignments were done with Basic Local Alignment Tool (BLAST) [89]. Searches for integrated phages were done using phaster [90]. CRISPRFinder were used to indentify CRISPRs, [91]. Prediction of genomic islands were done using Island Viewer 4 [92]. Five or four genes evenly distributed along the length of Gi-I or II respectively, were selected and used as a query for a blastn search against the nucleotide collection of the NCBI, excluding uncultured organisms. The two best hits, when applicable, were used to get an indication of the phylogenetic association of the GI.

## Additional files

Additional file 1: **Table S1.** Average nucleotide identity (ANI); **Figure S1.** Pairwise synteny dot plots; **Figure S2.** Organization of CRISPR clusters; **Figure S3.** Circular representation of the genome of “*Ca*. Methylacidiphilum fumariolicum” SolV; **Figure S4.** Circular representation of the genome of “*Ca*. Methylacidiphilum infernorum” V4; **Table S2.** Result of Blastn searches of selected genes from genomic island I and II; **Table S3.** Genes associated with methane oxidation; **Table S4.** *pmoD* homologs; **Table S5**, A-D). Similarity (bottom) and identity (top) matrix generated from the amino acid sequence of the PmoA, B, C and Ds; **Figure S5.** Genome alignment of strains Kam1, SolV and V4, showing the region encoding *pmo* cluster I; **Figure S6.** Genome alignment of strains Kam1, SolV and V4, showing the region encoding *pmo* cluster II; **Figure S7.** Genome alignment of strains Kam1, SolV and V4, showing the region encoding *pmo* cluster III; **Table S6.** Genes associated with CO_2_ fixation; **Table S7.** Genes associated with glycogen metabolism; **Table S8.** Genes associated with hydrogenases; **Figure S8.** Genome alignment of strains Kam1, SolV and V4, showing the region encoding the type 1h hydrogenase; **Table S9.** Genes associated with nitrogen metabolism; **Figure S9.** Genome alignment of strains Kam1, SolV and V4, showing the regions encoding putative NirKs in strain SolV; **Table S10.** Genes encoding components of electron transport chains; **Figure S10.** Organization of the two *atp* operons; **Table S11.** Genes speculated to be involved in acid resistance; **Table S12.** Genes predicted to have a role in heavy metal resistance. (DOCX 3318 kb)

Additional file 2: Genome coordinates of the features shown on the outer ring of Fig. 1 and Additional file 1: Figures S3-S4. (XLSX 16 kb)

## Data Availability

The annotated complete genome of “*Ca*. Methylacidiphilum kamchatkense” Kam1 is deposited at Genbank under accession: CP037899 and at the Integrated Microbial Genomes and Microbiomes IMG/ER server under the genome ID: 2770939480.

## Abbreviations

ACIII: Alternative complex III
ADAR: Arginine-dependent acid resistance
ANI: Average nucleotide identity
BLAST: Basic Local Alignment Tool
BRIG: Blast Ring Image Generator
*Ca*.: *Candidatus*
CBB: Calvin Benson Bassham
Ehr: Ech hydrogenase related
GDAR: Glutamic acid dependent acid resistance
GI: Genomic island
MATGAT: Matrix Global Alignment tool
MDH: Methanol dehydrogenase
MK: Menaquinone
NCBI: National Center for Biotechnology Information
pMMO: Particulate methane monooxygenase
sMMO: Solouble methane monooxygenase
Tat: Twin arginine translocation
TCA: Tricarboxylic acid cycle
TE: Transposable elements
