# Supplementary material for: Complete genome sequence analysis of the thermoacidophilic verrucomicrobial methanotroph “Candidatus Methylacidiphilum kamchatkense” strain Kam1 and comparison with its closest relatives
Source: BMC Genomics. 2019 Aug 9;20:642. doi: 10.1186/s12864-019-5995-4 (PMC6688271; doi:10.1186/s12864-019-5995-4)
Supplement: Supplementary file 1 — Table S1. Average nucleotide identity (ANI); Figure S1. Pairwise synteny dot plots; Figure S2. Organization of CRISPR clusters; Figure S3. Circular representation of the genome of “Ca. Methylacidiphilum fumariolicum” SolV; Figure S4. Circular representation of the genome of “Ca. Methylacidiphilum infernorum” V4; Table S2. Result of Blastn searches of selected genes from genomic island I and II; Table S3. Genes associated with methane oxidation; Table S4. pmoD homologs; Table S5, A-D). Similarity (bottom) and identity (top) matrix generated from the amino acid sequence of the PmoA, B, C and Ds; Figure S5. Genome alignment of strains Kam1, SolV and V4, showing the region encoding pmo cluster I; Figure S6. Genome alignment of strains Kam1, SolV and V4, showing the region encoding pmo cluster II; Figure S7. Genome alignment of strains Kam1, SolV and V4, showing the region encoding pmo cluster III; Table S6. Genes associated with CO2 fixation; Table S7. Genes associated with glycogen metabolism; Table S8. Genes associated with hydrogenases; Figure S8. Genome alignment of strains Kam1, SolV and V4, showing the region encoding the type 1h hydrogenase; Table S9. Genes associated with nitrogen metabolism; Figure S9. Genome alignment of strains Kam1, SolV and V4, showing the regions encoding putative NirKs in strain SolV; Table S10. Genes encoding components of electron transport chains; Figure S10. Organization of the two atp operons; Table S11. Genes speculated to be involved in acid resistance; Table S12. Genes predicted to have a role in heavy metal resistance. (DOCX 3318 kb) [file 12864_2019_5995_MOESM1_ESM.docx]

**Table S1.** Average nucleotide identity (ANI) calculated with the JSpecies V 1.2.1 software [1]. Results are given in columns with the strain designation of the subject given on top. Strains with an ANI > 95% is considered as belonging to the same species. Methylacidiphilum is abbreviated M.

|  | **Kam1** | **SoIV** | **V4** |
| --- | --- | --- | --- |
| ***"Ca.* M. kamchatkense” Kam1** | --- | 92.54 | 71.65 |
| ***"Ca.* M. fumariolicum” SoIV** | 92.38 | --- | 71.74 |
| ***"Ca.* M. infernorum” V4** | 71.61 | 71.67 | --- |


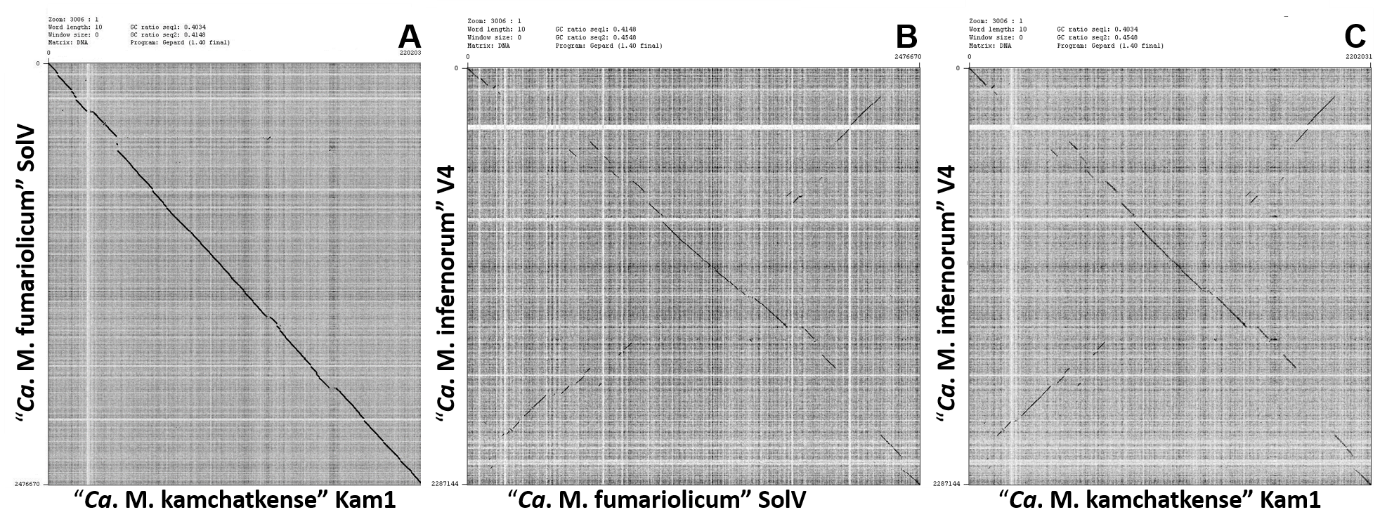


**Figure S1.** Pairwise synteny dot plot comparisons of strains Kam1, SolV and V4 as indicated on the axis of the plots. Methylacidiphilum is abbreviated M. A straight diagonal line from corner to corner would signify 100 % synteny between the two genomes, 90° deviations indicates inversions of genome segments. Plots were constructed with Gepard v 1.40 [2].


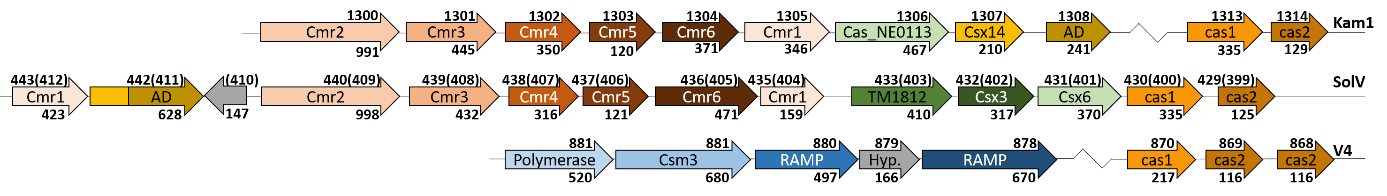


**Figure S2.** Organization of the CRISPR clusters of strains Kam1, SolV and V4. Figure is based on the annotation available at the joint genome institute and is not drawn to scale. Strain designations are given to the right, locus tags without prefix are given on top of arrows, for strain SolV locus tags without or between brackets stems from Mohammadi and colleagues 2017 [3] or Integrated Microbial genomes and Microbiomes IMG/ER [4] respectively (see table 1). Number of encoded amino acids are given below arrows. Homologous genes are given the same color; hypothetical genes are indicated with grey. RAMP: Repeat Associated Mysterious Proteins; Cmr: Cas module-RAMP; AD: adenosine deaminase; TM1812: CRISPR-assoc_prot_TM1812; Polymerase: CRISPR associated polymerase. Zigzag line indicates stretch of DNA with no predicted role in CRISPR.


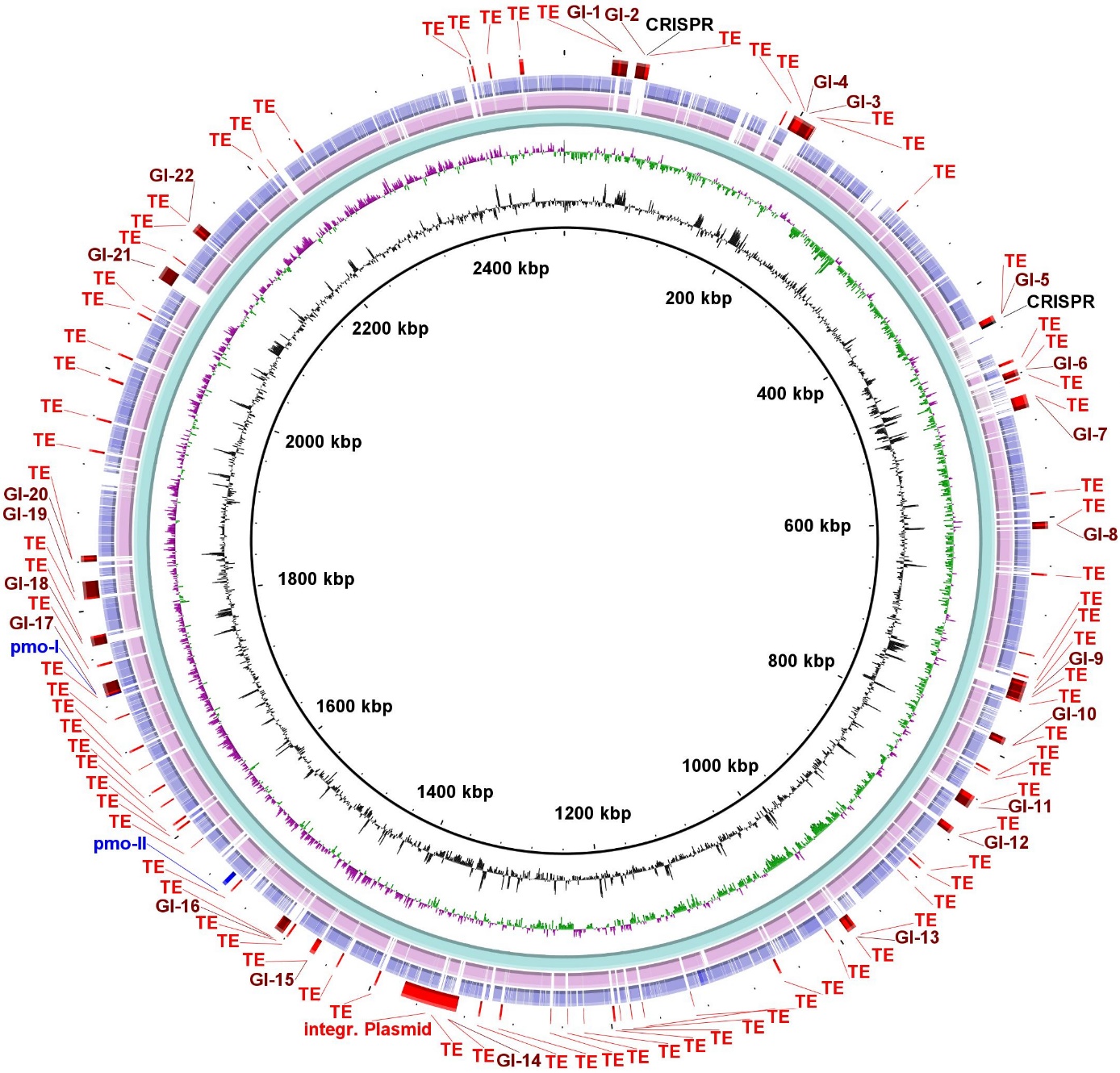


**Figure S3.** Circular representation of the genome of “*Ca*. Methylacidiphilum fumariolicum” SolV. Rings from inside to outside: 1) GC content (black); 2) GC skew (-/+ purple/green); 3) strain SolV; 4) strain Kam1; 5) strain V4; 6) Selected genomic traits of strain SolV for exact genomic coordinates see (Additional file 2). GI: Genomic island; TE: Transposable element; Pmo: *pmo* cluster. Figure were generated with Blast Ring Image Generator (BRIG) [5].


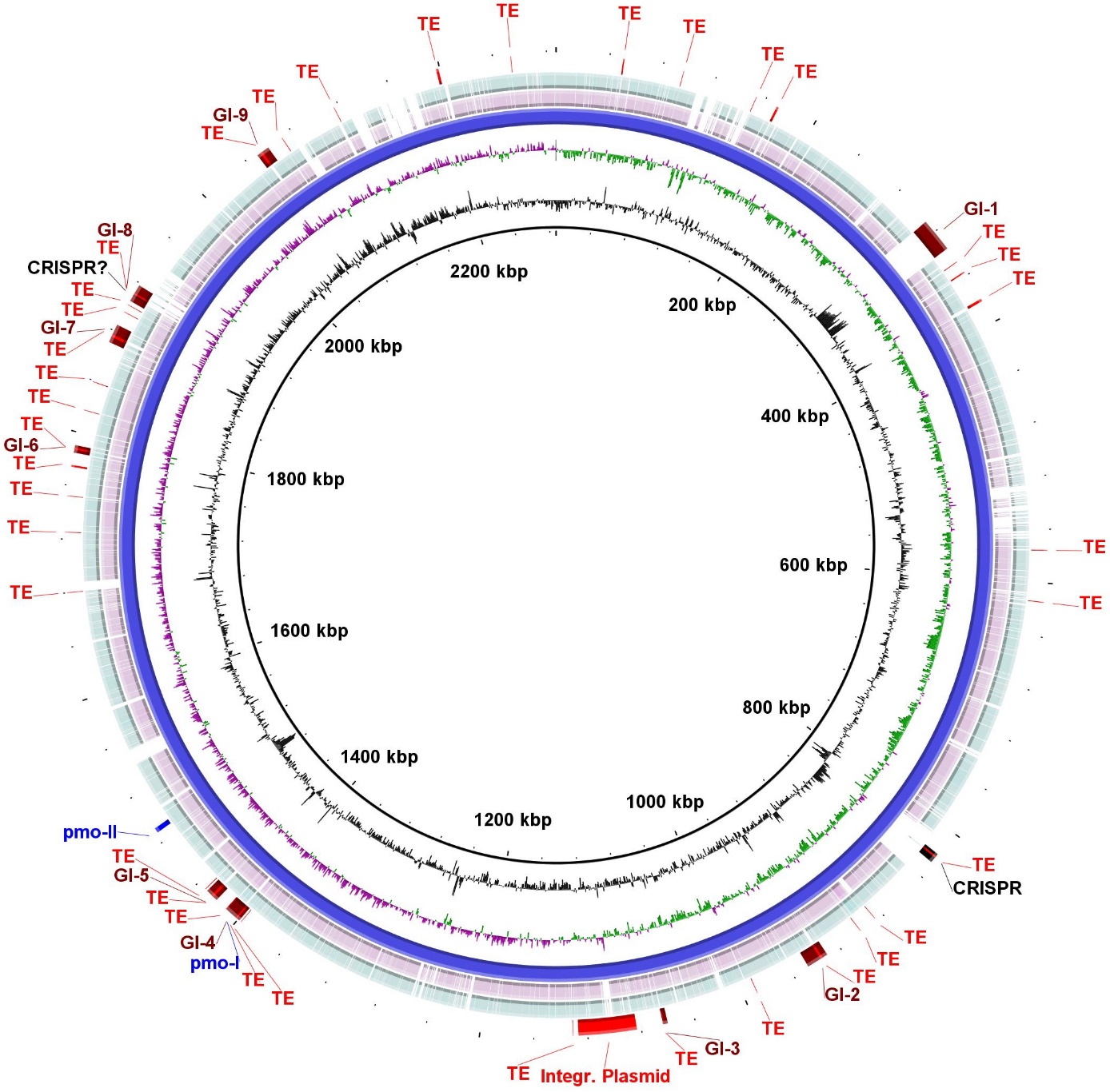


**Figure S4.** Circular representation of the genome of “*Ca*. Methylacidiphilum infernorum” V4. Rings from inside to outside: 1) GC content (black); 2) GC skew (-/+ purple/green); 3) strain V4; 4) strain Kam1; 5) strain SolV; 6) Selected genomic traits of strain V4 for exact genomic coordinates see (Additional file 2). GI: Genomic island; TE: Transposable element; Pmo: *pmo* cluster. Figure were generated with Blast Ring Image Generator (BRIG) [5].

**Table S2.** Result of Blastn searches of selected genes from genomic island I and II. Five genes taken from the entire length of genomic Island I and all four predicted genes from II were used as query for tblastn searches against the NCBI database (7-3-2019). E values > 0,0005 were omitted. Locus tags are given without prefix, see also (Table 1).

**Table S3.** Genes associated with methane oxidation in strains Kam1, SolV and V4. Strain designations are given on top of columns, and locus tags are given without prefix, for full species names and locus tags see (Table 1). ^*^ Locus tags following Mohammadi and colleagues 2017 [3]. ^#^ locus tags from Integrated Microbial genomes and Microbiomes IMG/ER [4]. In those instances where no gene had been annotated but an open reading frame could be identified the location on the forward strand is given between brackets (..), c indicates that the gene is located in the reverse strand. Locus tags of genes spanning two or more openreading frames, due to mutations, are separated with a hyphen -. █: Gene contains a frameshift mutation, that is not observed in the draft genome of strain Kam1 [6].

**Table S4.** *pmoD* homologs encoded on the genomes of strains Kam1, SolV and V4. Locus tags are given without prefix, for strain SolV locus tags without or between brackets stems from Mohammadi and colleagues 2017 [3] or Integrated Microbial genomes and Microbiomes IMG/ER [4] respectively, (see table 1). * No gene had been annotated but an open reading frame could be identified the location on the forward strand is given between brackets (..), c indicates that the gene is located in the reverse strand. TMH: transmembrane helices.

**Table S5, A-D).** Similarity (bottom) and identity (top) matrix generated from the amino acid sequence of the PmoA, B, C and Ds, encoded on the genomes of strains Kam1, SolV and V4. Locus tags without prefix followed by strain designation are given at the top of each column and at the start of each row (For locus tags prefixes see (Table 1)). For strain SolV locus tags without or between brackets refers to the annotation following Mohammadi and colleagues 2017 [3] or Integrated Microbial genomes and Microbiomes IMG/ER [4] respectively, see also (Table 1). Calculations were done using using MatGAT with default settings [7]. **D)** * Gene not annotated but an open reading frame was identified on the complementary strand at base 1487863 to 1488489, forward strand numbering. CRISPR: genes are found in a CRISPR region. Cu: Genes found next to a copC gene (Kam1 and SolV) or a glycosyl transferase (V4).

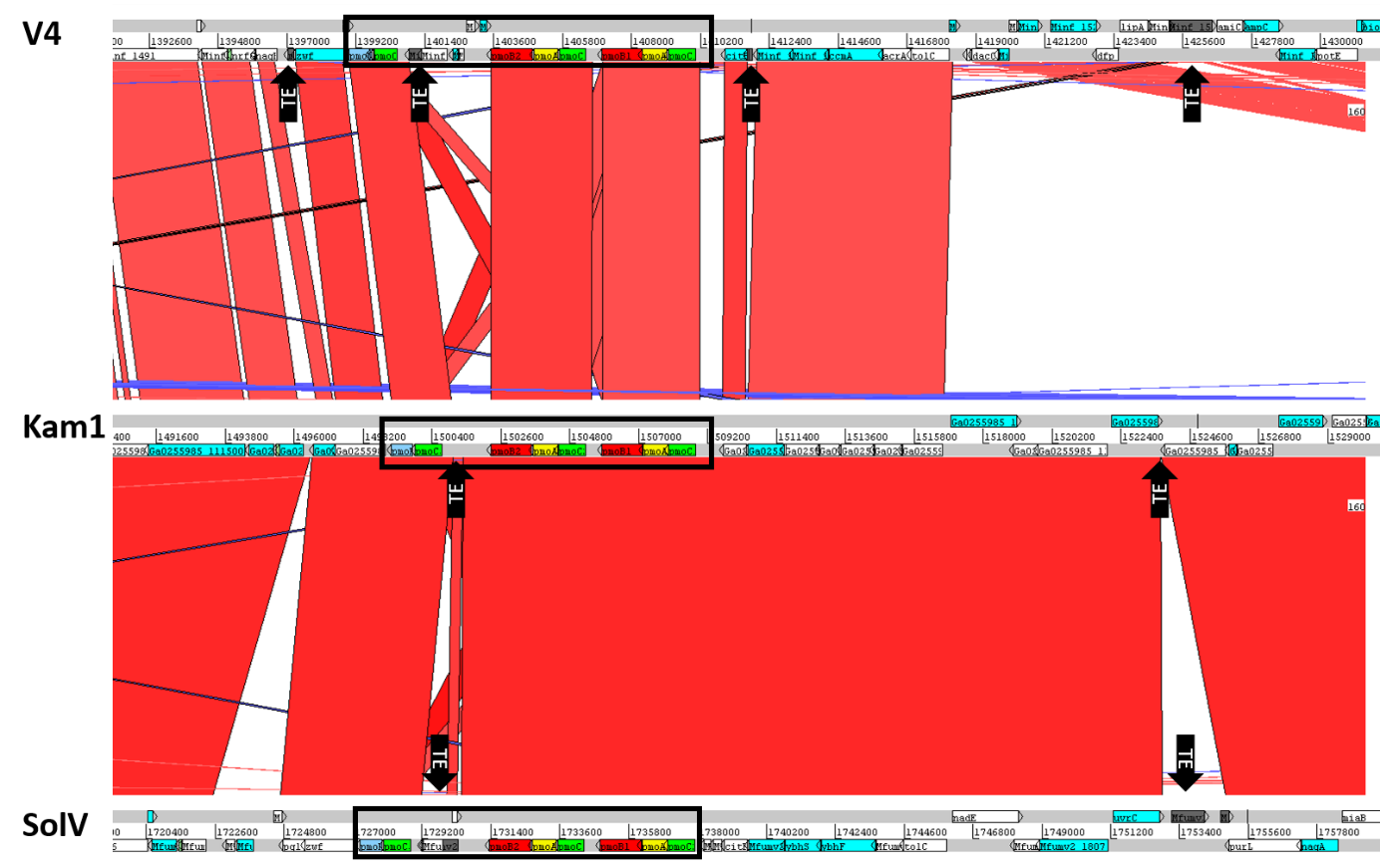


**Figure S5.** Genome alignment of strains Kam1, SolV and V4, showing the region encoding *pmo* cluster I, highlighted with a black box. Strain designations without genus or species names are shown to the left. For full species names and locus tags see (Table 1). Filled areas between genomes, indicate conserved regions, red is in the same order, whereas blue means inverse order. Predicted transposable elements (TE) are indicated with black arrows. Figure were generated with ACT v13 [8].


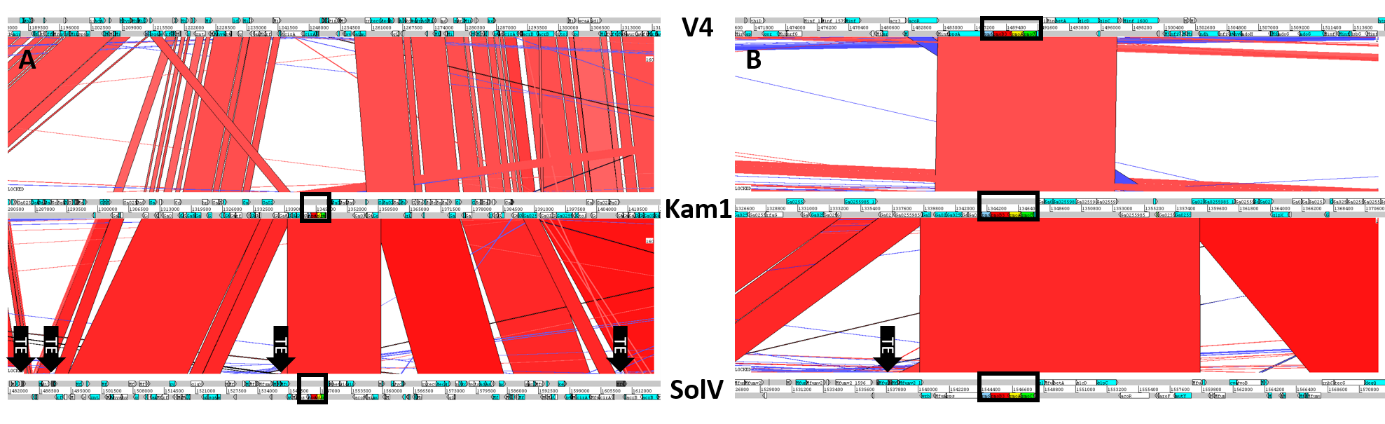


**Figure S6.** Genome alignment of strains Kam1, SolV and V4, showing the region encoding *pmo* cluster II, highlighted with a black box. **A)** Alignment centered on the flanking regions of pmo cluster II. **B)** Alignment centered on pmo cluster II. Strain designations without genus or species names are shown between alignments. For full species names and locus tags see (Table 1). Filled areas between genomes, indicate conserved regions, red is in the same order, whereas blue means inverse order. Predicted transposable elements (TE) are indicated with black arrows. Figure were generated with ACT v13 [8].


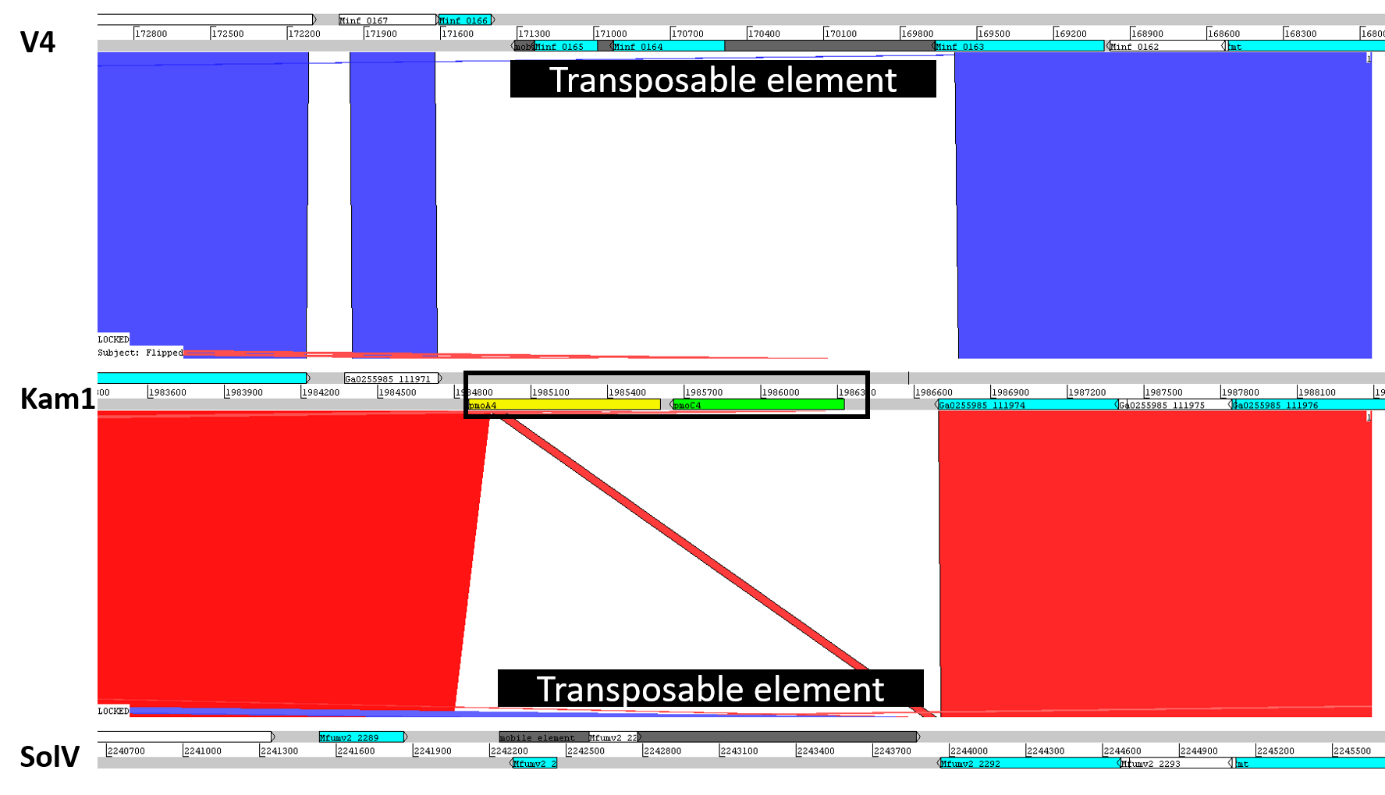


**Figure S7.** Genome alignment of strains Kam1, SolV and V4, showing the region encoding *pmo* cluster III, highlighted with a black box. Strain designations without genus or species names are shown to the left of the genomes. For full species names and locus tags see (Table 1). Filled areas between genomes, indicate conserved regions, red is in the same order, whereas blue means inverse order. Figure were generated with ACT v13 [8].

**Table S6.** Genes associated with CO_2_ fixation in strains Kam1, SolV and V4. Strain designations are given on top of columns, and locus tags are given without prefix, for full species names and locus tags see (Table 1). ^*^ Locus tags following Mohammadi and colleagues 2017 [3]. ^#^ Locus tags from Integrated Microbial genomes and Microbiomes IMG/ER [4]. Locus tags separated with a slash “/” indicates that more genes are annotated as having the same function. EC #: Enzyme commission number.

**Table S7.** Genes associated with glycogen metabolism in strains Kam1, SolV and V4. Strain designations are given on top of columns, and locus tags are given without prefix, for full species names and locus tags see (Table 1). ^*^ Locus tags following Mohammadi and colleagues 2017 [3]. ^#^ Locus tags from Integrated Microbial genomes and Microbiomes IMG/ER [4]. Locus tags of genes spanning two open reading frames, due to mutations, are separated with a hyphen “-“. █: Gene contains a frameshift mutation, that is not observed in the draft genome of strain Kam1 [6]. Locus tags separated with a slash “/” indicates that more genes are annotated as having the same function

**Table S8.** Genes associated with hydrogenases in strains Kam1, SolV and V4. Strain designations are given on top of columns, and locus tags are given without prefix, for full species names and locus tags see (Table 1). ^*^ Locus tags following Mohammadi and colleagues 2017 [3]. ^#^ Locus tags from Integrated Microbial genomes and Microbiomes IMG/ER [4]. ^$^ C terminal truncated. Locus tags of genes spanning two open reading frames, due to mutations, are separated with a hyphen “-“. █: Gene contains a frameshift mutation, that is not observed in the draft genome of strain Kam1 [6].

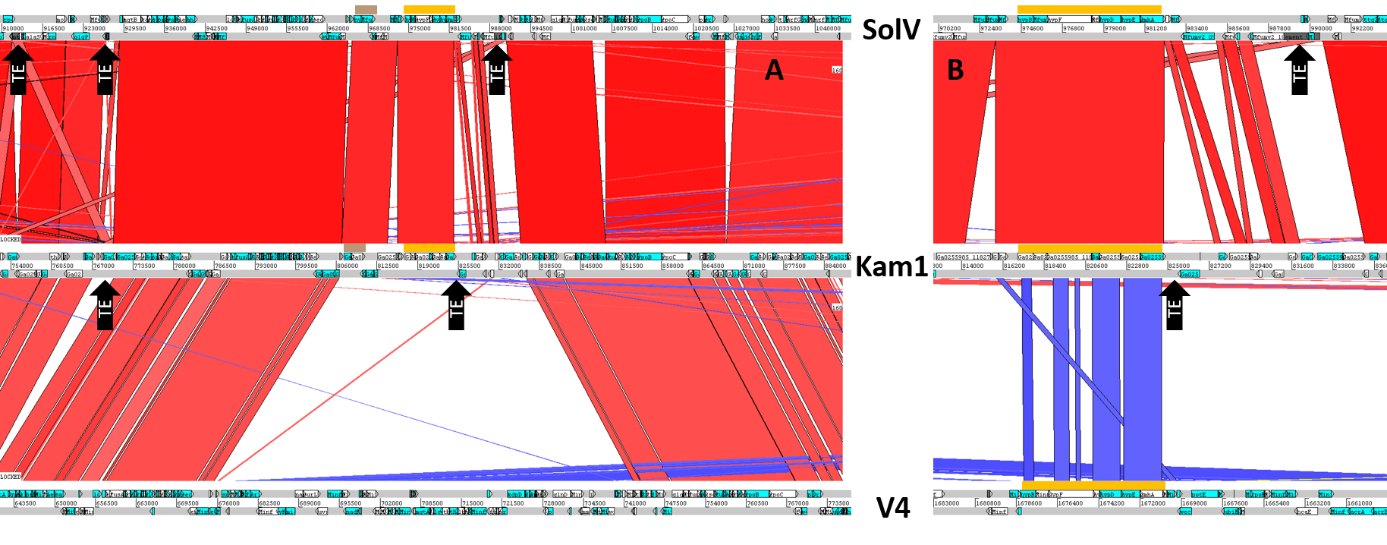


**Figure S8.** Genome alignment of strains Kam1, SolV and V4, showing: The region encoding the type 1h hydrogenase, *hhySL*, indicated with a brown bar: The genes encoding [NiFe] hydrogenase metallocenter assembly proteins, *hypFCDE*, is indicated with an orange bar. **A)** Alignment centered on the flanking regions of the *hypFCDE*, genes. **B)** Alignment centered on the *hypFCDE*, genes. Strain designations without genus or species names are shown between the alignments. For full species names and locus tags see (Table 1). Filled areas between genomes, indicate conserved regions, red is in the same order, whereas blue means inverse order. Predicted transposable elements (TE) are indicated with black arrows. Figure were generated with ACT v13 [8].

**Table S9.** Genes associated with nitrogen metabolism in strains Kam1, SolV and V4. Strain designations are given on top of columns, and locus tags are given without prefix, for full species names and locus tags see (Table 1). ^*^ Locus tags following Mohammadi and colleagues 2017 [3]. ^#^Locus tags from Integrated Microbial genomes and Microbiomes IMG/ER [4]. In one instance where no gene had been annotated, but an open reading frame could be identified the base range on the forward strand is given as numbers separated by “..”. Locus tags of genes spanning three open reading frames, due to mutations, are separated with a hyphen “-“, █: Gene contains a frameshift mutation, that is not observed in the draft genome of strain Kam1 [6]

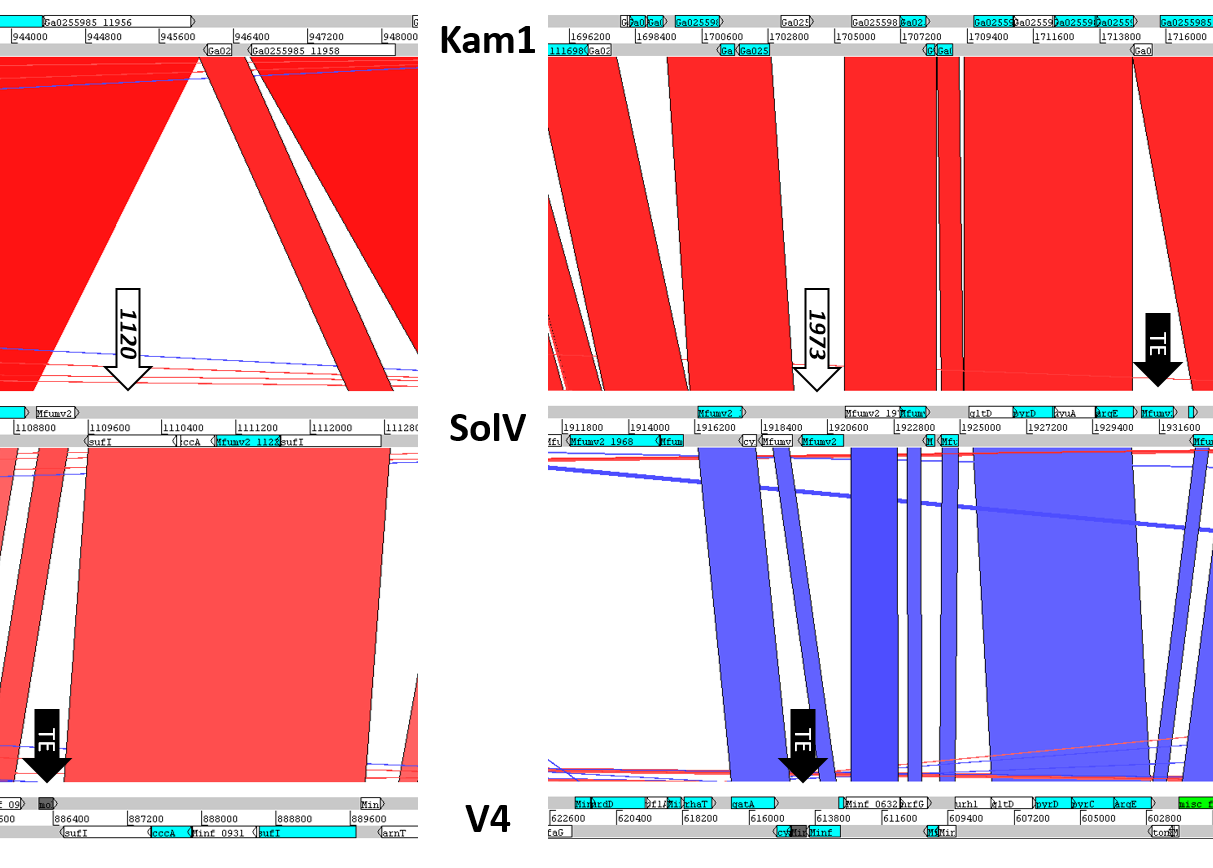


**Figure S9.** Genome alignment of strains Kam1, SolV and V4, showing the regions encoding putative NirKs in strain SolV. Location of the *nirK* genes are indicated with a white arrow. Numbers within arrows are locus tags are given without prefix following Mohammadi and colleagues 2017 [3] see also (Table 1 and Additional file 1: Table S9). Predicted transposable elements (TE) are indicated with black arrows. Strain designations without genus or species names are shown between alignments. For full species names and locus tags see (Table 1). Filled areas between genomes, indicate conserved regions, red is in the same order, whereas blue means inverse order. Figure were generated with ACT v13 [8].

**Table S10.** Genes encoding components of the electron transport chain, from strains Kam1, SolV and V4. Strain designations are given on top of columns, and locus tags are given without prefix, for full species names and locus tags see (Table 1). ^*^ Locus tags following Mohammadi and colleagues 2017 [3]. ^#^ Locus tags from Integrated Microbial genomes and Microbiomes IMG/ER [4]. Locus tags of genes spanning two or more open reading frames, due to mutations, are separated with a hyphen “-“. █: Gene contains a frameshift mutation, that is not observed in the draft genome of strain Kam1 [6]. Locus tags separated with an slash “/,” indicates that more genes are annotated as having the same function.

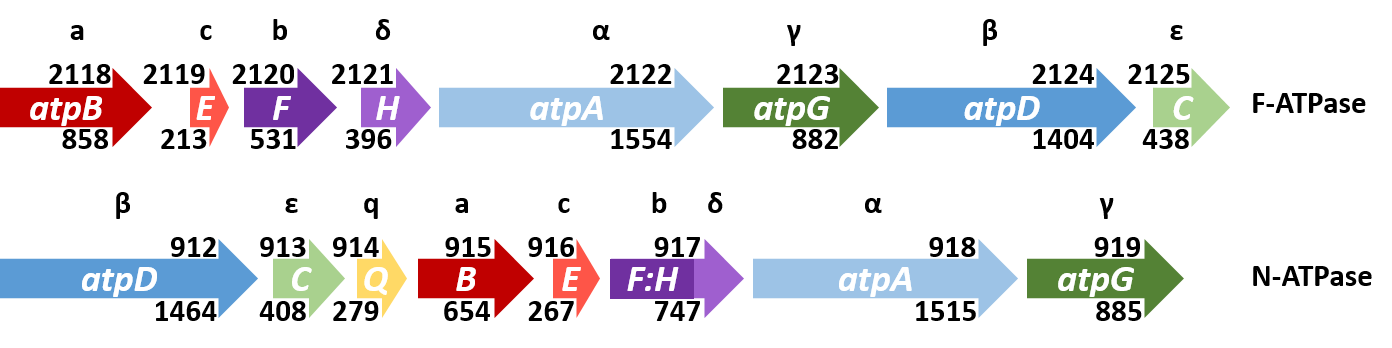


**Figure S10.** Organization of the two *atp* operons from “*Ca*. Methylacidiphilum kamchatkense” Kam1. Numbers on top of arrows indicates locus tags without prefix, see (Table 1) for locus tags. Numbers under arrows indicates gene size in bp. Symbols above arrows indicate gene products. Gene names are given within arrows, for practical reasons the *atp* prefix is left out for short genes.

**Table S11.** Genes speculated to be involved in acid resistance of strains Kam1, SolV and V4. Strain designations are given on top of columns, and locus tags are given without prefix, for full species names and locus tags see (Table 1). ^*^ Locus tags Mohammadi and colleagues 2017 [3]. ^#^ Locus tags from Integrated Microbial genomes and Microbiomes IMG/ER [4]. Locus tags of genes spanning two or more open reading frames, due to mutations, are separated with a hyphen “-“. █: Gene contains a frameshift mutation, that is not observed in the draft genome of strain Kam1 [6].

**Table S12.** Genes predicted to have a role in heavy metal resistance of strains Kam1, SolV and V4. Strain designations are given on top of columns, and locus tags are given without prefix, for full species names and locus tags see (Table 1). ^*^ Locus tags following Mohammadi and colleagues 2017 [3]. ^#^ locus tags from Integrated Microbial genomes and Microbiomes IMG/ER [4]. In one instance were no gene had been annotated but an open reading frame could be identified the location on the forward strand is given between brackets (..), c indicates that the gene is located in the reverse strand. Locus tags of genes spanning two or more open reading frames, due to mutations are separated with a hyphen “-“. █: Gene contains a frameshift mutation, that is not observed in the draft genome of strain Kam1. █: Gene contains a frameshift mutation, that is not observed in the draft genome of strain Kam1, but the gene in the draft genome contains a frameshift mutation at another location [6].

1. Richter M, Rossello-Mora R: **Shifting the genomic gold standard for the prokaryotic species definition**. *Proceedings of the National Academy of Sciences of the United States of America* 2009, **106**(45):19126-19131.

2. Krumsiek J, Arnold R, Rattei T: **Gepard: a rapid and sensitive tool for creating dotplots on genome scale**. *Bioinformatics (Oxford, England)* 2007, **23**(8):1026-1028.

3. Mohammadi S, Pol A, van Alen TA, Jetten MS, Op den Camp HJ: ***Methylacidiphilum fumariolicum* SolV, a thermoacidophilic 'Knallgas' methanotroph with both an oxygen-sensitive and -insensitive hydrogenase**. *Isme j* 2017, **11**(4):945-958.

4. Chen IA, Chu K, Palaniappan K, Pillay M, Ratner A, Huang J, Huntemann M, Varghese N, White JR, Seshadri R *et al*: **IMG/M v.5.0: an integrated data management and comparative analysis system for microbial genomes and microbiomes**. *Nucleic acids research* 2019, **47**(D1):D666-d677.

5. Alikhan N-F, Petty NK, Ben Zakour NL, Beatson SA: **BLAST Ring Image Generator (BRIG): simple prokaryote genome comparisons**. *BMC genomics* 2011, **12**:402-402.

6. Erikstad HA, Birkeland NK: **Draft Genome Sequence of "*Candidatus* Methylacidiphilum kamchatkense" Strain Kam1, a Thermoacidophilic Methanotrophic Verrucomicrobium**. *Genome announcements* 2015, **3**(2).

7. Campanella JJ, Bitincka L, Smalley J: **MatGAT: An application that generates similarity/identity matrices using protein or DNA sequences**. *BMC Bioinformatics* 2003, **4**(1):29.

8. Carver TJ, Rutherford KM, Berriman M, Rajandream M-A, Barrell BG, Parkhill J: **ACT: the Artemis comparison tool**. *Bioinformatics (Oxford, England)* 2005, **21**(16):3422-3423.
